# Executive Impairment in Huntington's Disease: Insights From a Systematic Review of the Literature

**DOI:** 10.1002/brb3.71238

**Published:** 2026-01-31

**Authors:** Simone Migliore, Martina Marcaccio, Ilaria Di Pompeo, Massimo Marano, Giuseppe Curcio

**Affiliations:** ^1^ Department of Biotechnological and Applied Clinical Sciences University of L'Aquila L'Aquila Italy; ^2^ Fondazione Policlinico Universitario Campus Bio‐Medico Rome Italy; ^3^ Unit of Neurology, Neurophysiology, Neurobiology and Psychiatry, Department of Medicine University Campus Bio‐Medico of Rome Rome Italy

**Keywords:** cognitive impairment, executive functioning, frontal lobe, Huntington's disease, neurodegeneration

## Abstract

**Purpose:**

This systematic review examines executive dysfunction in Huntington's disease (HD), an inherited neurodegenerative disorder characterized by cognitive alterations that may emerge years before the onset of motor symptoms. The objective of this review is to provide an updated and comprehensive synthesis of executive function deficits observed across the clinical spectrum of HD—from presymptomatic to the more advanced symptomatic phases—and to examine how these deficits relate to underlying neurobiological changes identified through neuroimaging and neurophysiological studies.

**Method:**

A systematic review was conducted encompassing studies published up to November 2024 and listed on PubMed database. Inclusion criteria focused on clinical and experimental studies involving executive functions in HD at different stages. A total of 3487 articles were screened, of which 115 met the eligibility criteria. The present review synthesizes recent longitudinal and multimodal findings, with a specific focus on the presymptomatic and prodromal phases, offering an integrative perspective that links cognitive, structural, and functional markers of executive dysfunction.

**Finding:**

The findings reveal a selective, stage‐dependent pattern of executive dysfunction. Early alterations are most consistently observed in psychomotor speed, cognitive flexibility, response inhibition, and working memory updating, whereas later stages exhibit broader impairments in planning and attention, accompanied by overall functional decline. Among neuroimaging markers, striatal atrophy, frontostriatal disconnection, and reduced prefrontal activation during executive tasks emerge as the most robust correlates of executive dysfunction.

**Conclusion:**

These results underscore the relevance of early cognitive assessment for detecting subtle executive changes prior to overt motor symptoms, and the need for longitudinal, multimodal and computational approaches integrating behavioral, neuroimaging, and electrophysiological data. Future research should prioritize prospective tracking of presymptomatic individuals, the standardization of executive function measures, and the identification of biologically markers to inform early interventions and monitor treatment efficacy.

## Graphical abstract

Executive dysfunction in Huntington's disease follows a selective, stage‐dependent pattern, with early deficits in psychomotor speed, cognitive flexibility, inhibition, and working‐memory updating. Progression is associated with broader impairments in planning and attention. These cognitive changes parallel striatal atrophy, frontostriatal disconnection, and reduced prefrontal activation. Early, multimodal assessment is crucial for detecting subtle executive alterations prior to motor onset.

DLPFC, dorsolateral prefrontal cortex; VLPFC, ventrolateral prefrontal cortex.

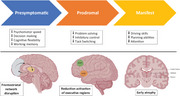

## 1 Introduction

Huntington's disease (HD) is an autosomal dominant inherited neurodegenerative disorder caused by the transmission of an abnormal expansion of the CAG trinucleotide repeat of the huntingtin gene (HTT) to the offspring. As a result, a polyglutamine tail alters the physiological huntingtin conformation (McColgan and Tabrizi 2018), resulting in a loss of physiological function with gain of toxic properties.

The syndrome includes variable symptoms encompassing motor alteration (e.g., chorea, dystonia, parkinsonism, impaired ocular movements, limb incoordination, and loss of balance) as well as neuropsychiatric symptoms (e.g., obsessions, perseveration, apathy, aggressiveness, depression, and propensity to suicide) and altered cognition (e.g., abnormal executive functions, memory, language, and social cognition; Bates et al. 2015).

Cognitive impairment frequently appears several years before the diagnosis of motor symptoms (Langley et al. 2021). Executive dysfunction represents one of the earliest and most disabling cognitive characteristics, affecting primarily planning, cognitive flexibility, working memory (WM), and inhibitory control (Papoutsi et al. 2014). These deficits, in combination with behavioral alterations, play a significant role to functional decline and loss of independence (Eddy et al. 2016; Migliore et al. 2024).

From the presymptomatic stage, atrophy in key regions—particularly the striatum and prefrontal cortex—disrupts frontostriatal circuits essential for executive control (Cristofori et al. 2019). Neuroimaging studies have demonstrated altered functional connectivity patterns that correlate with the severity of executive dysfunction (Domínguez D et al. 2017; Langley et al. 2021). A systematic review (Pini et al. 2020) confirmed progressive connectivity breakdown, from early sensory‐motor alterations to later frontoparietal disconnection, with reduced integration among frontal and striatal regions predicting poorer executive performance. These findings highlight dysfunctional prefrontal–subcortical communication as a major substrate of executive impairment in HD (Cristofori et al. 2019; Pini et al. 2020). This updated framework also situates executive dysfunction in HD within the broader spectrum of neurodegenerative disorders such as Parkinson's disease (PD) and frontotemporal dementia (FTD), which share frontostriatal and frontoparietal network vulnerability but differ in the timing and uniformity of executive decline. This comparison underscores HD as a model condition for studying frontostriatal degeneration and mechanisms of cognitive control.

The present review extends previous syntheses by systematically including studies published up to November 2024, with specific attention to presymptomatic and prodromal phases, and by integrating behavioral, neuroimaging, and neurophysiological data to identify converging markers of executive impairment. In summary, the aim of this review is to provide an updated, cross‐modal, and stage‐specific synthesis of executive dysfunction in HD and its neurobiological underpinnings.

## 2 Methods

The current systematic review was carried out based on the guidelines and principles outlined by the preferred reporting items for systematic reviews and meta‐analyses (PRISMA) statement 2020 and checklist (Page et al. 2021).

### 2.1 Search Strategy and Study Selection

We conducted a PubMed and PsycINFO search for studies published up to November 2024 using the following keywords: Huntington's disease AND (executive function* OR working memory OR cognitive flexibility OR monitoring OR switching OR response inhibition OR control inhibition OR planning OR problem solving OR attention OR attent* OR decision making). Only English language, original peer‐reviewed research articles were included.

### 2.2 Inclusion and Exclusion Criteria

Studies were included if they met predetermined inclusion criteria, that is, to be experimental studies carried out on executive functioning in HD subjects. For this review, executive functions were defined as core cognitive domains including WM, cognitive flexibility (set shifting), inhibitory control, verbal fluency, decision making, planning, information processing speed, and abstract reasoning. To maintain a specific focus on classic executive functions, studies centered on other constructs (e.g., social cognition or theory of mind) were excluded. Both manifest (symptomatic) HD patients and presymptomatic HD gene‐expansion carriers (pre‐HD) were considered to examine executive dysfunction across disease stages. The stage of HD was calculated according to the total motor score (TMS) and to the diagnostic confidence level (DCL). The section includes studies on presymptomatic (preHD, TMS < 10 and DCL < 4) and manifest HD (TMS ≥ 10 and DCL = 4). Pre‐HD subjects were classified, when possible, into preclinical (0 ≤ TMS ≤ 5) or prodromal (6 ≤ TMS ≤ 9). Available comparisons are reported, along with association studies examining the relationship between executive functions and neurophysiological and/or neuroimaging measures.

Finally, experimental studies that investigated executive functioning by using neuroimaging and neurophysiological techniques (i.e., electroencephalography—EEG; event‐related potentials—ERP; magnetoencephalography—MEG; positron emission tomography—PET; magnetic resonance imaging—MRI; functional magnetic resonance imaging—fMRI; transcranial magnetic stimulation—TMSs) were also included.

A stepwise screening process was applied. During identification, reviews, meta‐analyses non‐English paper, and studies unrelated to executive functions were excluded. In the screening phase, we excluded studies lacking sufficient information to determine the executive domain studied, the tools employed, participants’ genetic status, or disease stage, as well as those with uncontrolled experimental design. At the eligibility stage, studies comparing HD with other neurological or psychiatric disorders (e.g., Alzheimer's disease, PD, Tourette's syndrome, and schizophrenia) were excluded to maintain population homogeneity and focus specifically on executive dysfunction across HD stages.

### 2.3 Data Extraction and Analysis

No formal risk‐of‐bias tools (e.g., Newcastle–Ottawa Scale or the Cochrane risk‐of‐bias instrument) were applied to the heterogeneity of study designs and outcomes. Instead, study quality was assessed through a structured and reproducible qualitative framework: each study was independently evaluated by two reviewers according to predefined domains (sample characterization and selection bias in recruitment, assessment validity, statistical control, incomplete outcome data and reporting transparency). Discrepancies were resolved through discussion and consensus; in the rare cases where both reviewers rated a study as weak, the study was retained for completeness but its findings were weighted qualitatively with caution during synthesis. Due to heterogeneity in design and sample size, a qualitative weighting approach was applied, giving greater interpretative weight to methodologically stronger studies.

## 3 Results

### 3.1 Literature Search

**FIGURE 1:**
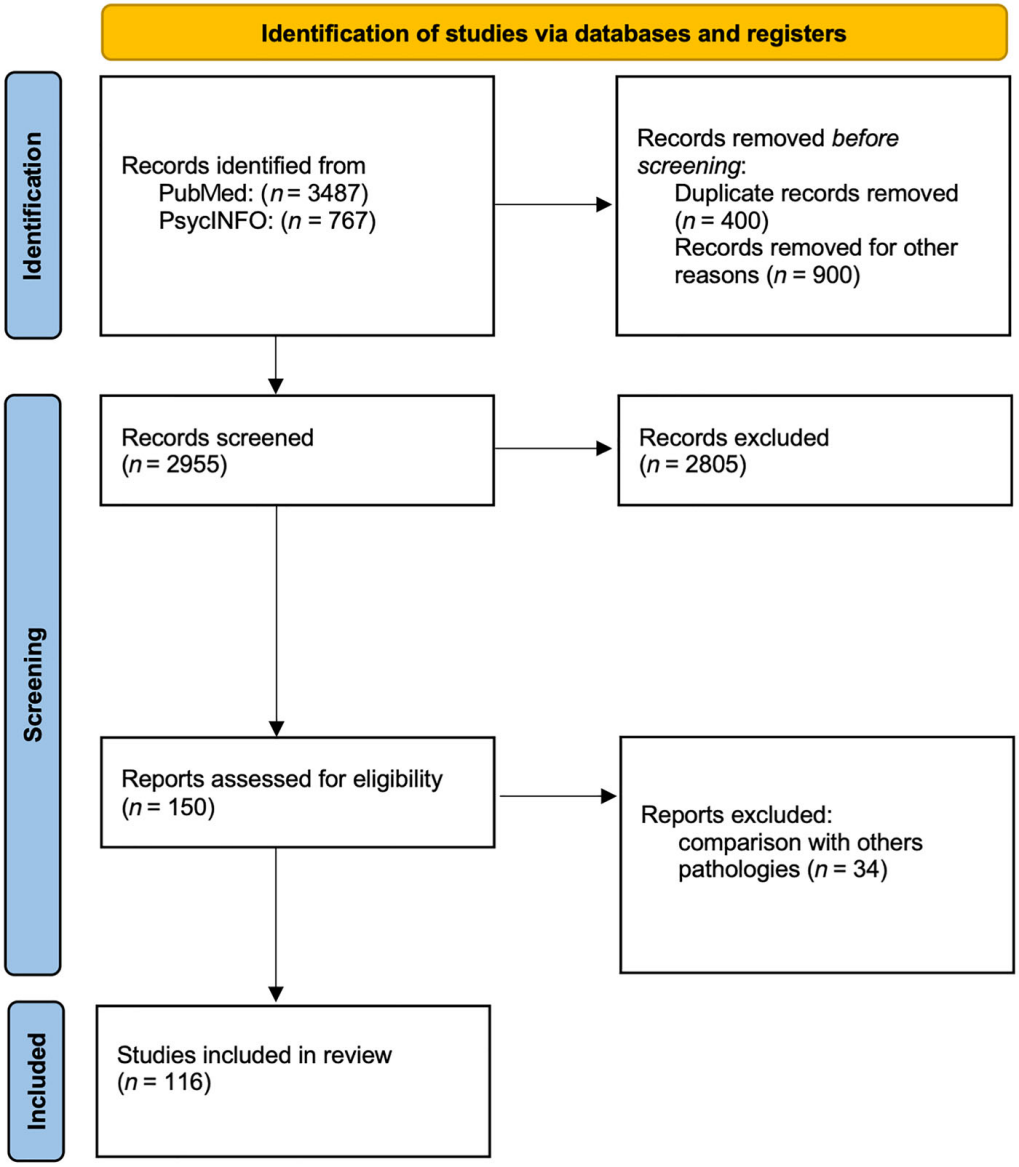
Preferred reporting items for systematic reviews and meta‐analyses (PRISMA) diagram of search strategy.

The database search identified 4254 records. In the initial phase, we removed reviews, systematic reviews, meta‐analyses, and studies that did not focus on executive functions. Furthermore, duplicates, articles not written in English were excluded, and thus only 2955 articles were screened for eligibility. After this step, 2805 articles were excluded because was not possible to identify the executive function being studied, the tools used, the genetic status, control or comparison group and/or to evaluate the disease stage of the patients. In the last step, 34 articles were excluded because they involved comparisons with other patient groups (Alzheimer's disease, Tourette's syndrome, Schizophrenia, and PD). The full text of the remained articles was examined. Thus, a total amount of 116 articles were included in the database and considered for the present review. Literature analysis and selection is summarized in Figure 1.

Each study has been analyzed and summarized in Table 1 (presymptomatic stage), Table 2 (manifest stage), or Table 3 (neuroimaging and neurophysiological methods for assessing executive functions in HD). Tables 1, 2, 3 show data on: participants and staging (i.e., premanifest, manifest), assessed cognitive domain with regard to executive functions, adopted measures and materials, main results, and the employed neuroimaging and/or neurophysiological technique if available.

**TABLE 1:** All studies related to the presymptomatic phase of the disease.

References	Participants	Cognitive domains assessed	Endpoint	Type of measure	Methodological comments	Results
1. Adjeroud et al. (2017)	23 HD (11 M; 50.3 ± 9.8) 20 pre‐HD (10 M; 36 ± 9) 16 HC for pre‐HD group (4 M; 42 ± 13.8) 23 HC for HD group (10 M; 50.8 ± 9.8)	Executive functions (attention shifting, working memory, learning and fine motor control, spontaneous and reactive flexibility)	Verbal fluency test, TMT, Stroop test, SDMT, Hopkins Verbal Learning Test, Mattis Dementia Rating Scale, IGT, GDT, The Barratt Impulsiveness Scale	Behavioral	Comparisons between groups	In this study emerges that pre‐HD vs. HC were unimpaired in performing executive tests as well as in decision‐making tasks, except for the Stroop task. HD were impaired in both the IGT and executive tasks, but not in the GDT. Decision‐making abilities are preserved during the premanifest stage of HD.
2. Brandt et al. (2008)	21 HD (8 M, 35.38 ± 10.13) 49 HD asymptomatic (23 M, 35.88 ± 9.76) 134 mutation‐negative (55 M, 37.15 ± 9.75)	Executive function, memory, language, processing speed	MMSE, WAIS‐R, Grooved Pegboard Test, SDMT written trial, Standardized Road‐Map Test of Directional Sense, HVLT, Brief Test of Attention, Stroop Color‐Word Test, WCST	Behavioral	Longitudinal study	Subjects with the Huntington's disease mutation who are carefully examined neurologically and found to be asymptomatic have very minimal problem‐solving impairment, and only if they are within a few years of clinical onset.
3. Campodonico et al. (1996)	22 pre‐HD (9 M, 31.9 ± 6.1) 37 gene negative (14 M, 35.8 ± 8.9)	Attention, memory, executive function	WAIS‐R, HVLT, SDMT, Stroop Color and Word Test, WCST, cognitive failures questionnaire, BDI, Beck Hopelessness scale, SCL‐90‐R, QNE	Behavioral	Longitudinal study	The study shows that subjects who are likely nearing clinical onset of HD may develop minor deficits in attention and mental speed before they reach threshold for diagnosis.
4. Carvalho et al. (2016)	215 HD low CAP 288 HD medium CAP 318 HD high CAP 233 HC	Executive functions (processing speed, working memory)	UHDRS, Neurobiological predictors of Huntington's disease, SDMT, TMT‐A, Stroop Color Naming, Word Reading, and Interference, Wechsler Abbreviated Scale of Intelligence, HVLT	Behavioral and oculomotor functions	Comparisons between groups	Participants with impaired oculomotor functioning performed worse than those with normal oculomotor functioning on cognitive tasks requiring oculomotor involvement, particularly on psychomotor speed tasks.
5. D'Aurizio et al. (2019)	30 pre‐HD (15 M, 34.32 ± 9.38) 21 HC (10 M, 33.67 ± 13.75)	Risky decision making	GDT, UHDRS	Behavioral	Comparisons between groups	In unfavorable and favorable decisions, preHD participants' decision and feedback times were longer than HC. Compared to HC, preHD subjects took longer to make decisions, indicating a lower propensity for risk.
6. Dumas et al. (2012)	120 pre‐HD 121 HD 122 HC	Visuospatial working memory	UHDRS, The Spot the Change task	Behavioral	Cross‐sectional study	Compared to controls, significant group differences in visuospatial working memory capacity (accuracy) were seen in PreHD and HD. These findings suggest that visuospatial working memory impairments are detectable in both pre‐HD and HD.
7. El Haj et al. (2023)	19 pre‐HD (11 M,45.32 ± 9.98) 23 HD (11 M, 52.04 ± 11.70) 23 HC (12 M,48.09 ± 7.15)	Decision making	UHDRS, MMSE, TMT, Stroop task, SDMT, Temporal discounting task	Behavioral	Comparisons between groups	Subjects with manifest HD exhibited higher temporal discounting than either pre‐HD or HC. Additionally, analysis also demonstrated significant correlations between temporal discounting and scores on an inhibition test in participants with manifest HD, but not in those with pre‐HD and HC.
8. Farrow et al. (2006)	16 pre‐HD (7 M, 35.6 ± 9.8) 24 HC (8 M, 36.0 ± 8.8)	Attention, memory	MMSE, BDI, motor task, cognitive task	Behavioral	Comparisons between groups	Pre‐HD individuals, close to the onset of Huntington's disease, were compared to HC in cognitive and motor tasks involving responses to visuospatial stimuli. Pre‐HD subjects made more errors than HC when required to provide a spatially incongruent response, suggesting difficulty inhibiting automatic responses.
9. Farrow et al. (2007)	13 pre‐HD (6 M, 36.6 ± 10.8) 17 HC (6 M, 37.8 ± 8.6)	Attention	MMSE, BDI, NART, Attentional Blink task, Inhibition of Return task, Vibrotactile task	Behavioral	Comparisons between groups	In pre‐HD and HC, attentional abilities were assessed using the attentional blink and the inhibition of return task. The results indicated possible alterations in cognitive inhibition of undesired responses and automatic inhibition of visual attention in individuals with preclinical HD nearing clinical onset.
10. Hart et al. (2013)	26 pre‐HD (35 ± 7) 19 HD (44 ± 14) 87 HC (42 ± 11)	Intelligence quotients, memory, executive functions	UHDRS, MMSE, WAIS‐R, Wechsler Memory Scale, CVLT, Boston Naming, Stroop test, SDMT, TMT, RT	Behavioral	Comparisons between groups Follow‐up	Over a 10‐year period, pre‐HD, HD, and HC participants were monitored, assessing four cognitive factors: “motor speed,” “global cognition,” “memory,” and “executive function.” The study's primary findings indicate that pre‐HD is linked to a gradual decline in memory and executive function, even in individuals distant from the anticipated onset of the disease.
11. Hart et al. (2014)	40 HD (13 M, 49 ± 10) 21 pre‐HD (8 M, 45 ± 9) 28 HC (13 M, 50 ± 8)	Attention, perceptual speed, motor speed, visual scanning, strategic reasoning, planning, ignore distractors	UHDRS, MMSE, BDI‐II, SDMT, figure fluency test	Behavioral	Comparisons between groups	The SDMT and the figure fluency test were administered to both pre‐HD and HD patients. The study revealed that motor impairments negatively influence SDMT performance. Isolating the cognitive component of this test unveiled cognitive decline only in the pre‐HD group, particularly in individuals nearing the anticipated clinical onset of the disease.
12. Heim et al. (2020)	16 pre‐HD NEAR (10 M, 39.7 ± 8.9) 13 pre‐HD FAR (4 M, 33.2 ± 5.1) 22 HD (9 M, 50.7 ± 9.9) 26 HC (17 M, 40.7 ± 11.0)	Decision making	UHDRS, MoCA, CSSRS, Apathy Evaluation Scale, PBA, Beads task, TMT, SDMT	Behavioral	Comparisons between groups	The samples were compared on different tests. In the beads task, HD patients gathered less information than all other groups. The NEAR group gathered less information than the FAR group and HC. There was no difference between the HC and the FAR group. In the TMT and the SDMT, HD patients were slower than all other groups but there were no other significant differences.
13. Horta‐Barba et al. (2021)	31 pre‐HD (40.8 ± 8): 16 preHD‐A 15 preHD‐B 165 HD (49.3 ± 11) 134 early–mild HD (51.3 ± 11) 37 HC (47.7 ± 11)	Arithmetic word‐problem solving	UHDRS, MMSE, PD‐CRS, HD‐WPA	Behavioral	Comparisons between groups	Pre‐HD subjects nearing clinical onset scored lower than those further from onset and HC. Additionally, both pre‐HD and early HD patients made more errors with increasing trial complexity compared to HC. Arithmetic problem‐solving deteriorates early in HD, linked to deficient frontal‐executive and mentalizing processes.
14. Jacobs et al. (2018a)	28 pre‐HD (15 M; 38.4 ± 8.3) 30 HD (16 M; 52.8 ± 10.5) 29 HC (11 M; 48.7 ± 11.0)	Driving skills	UHDRS, Driving simulator, driving history questionnaire	Behavioral	Comparisons between groups	In this study HD drove slower compared HC and pre‐HD when speed limits increased and they had a less steady speed compared to pre‐HD on the motorway. HD had a larger standard deviation of the lateral position compared to HC and pre‐HD on the motorway.
15. Larsson et al. (2008)	29 pre‐HD (16 M, 36.6 ± 8.6) 34 HC (13 M, 38.0 ± 10.4)	Verbal fluency, memory, attention, executive function, working memory	The phonemic fluency test, the semantic fluency task, WAIS–R, Digit Span Backward, R 11 KB Codifying, Digit Symbol, Dots, WCST	Behavioral	Comparisons between groups	From the study, it emerges that compared to HC, pre‐HD produced fewer words and produced them more slowly in the phonemic fluency task but not in the semantic fluency task. Pre‐HD near the HD onset performed worse than HC on both fluency tasks.
16. Maurage et al. (2017)	18 pre‐HD (11 M: 40.4 ± 14.3) 20 HD (12 M; 49.1 ± 11.3) 18 HC_1 (11 M; 39.4 ± 15.0) 20 HC_2 (12 M; 49.6 ± 15.7)	Attention, executive control	Attentional networks task: alerting, orienting, and executive control	Behavioral	Comparisons between groups	Manifest HD is associated with a specific impairment for executive control, while alerting and orienting are preserved. No deficits for attentional networks were observed in pre‐HD, suggesting that attentional deficits might differentiate the successive stages of HD.
17. Migliore et al. (2019)	15 pre‐HD1 (10 M; 32.38 ± 7.62) 15 pre‐HD2 (5 M; 36.26 ± 10.78) 18 HC (10 M; 31.67 ± 13.1)	Executive functions (executive control, attentional shifting, engagement and disengagement of attention, task‐set reconfiguration ability)	Task‐switching protocol	Behavioral	Comparisons between groups.	Pre‐HD subjects had worse performance than HC in the switch and repetition trials, as indicated by increased SC and reaction times. Pre‐HD2 showed impaired switching abilities with reaction times slower than pre‐HD1 and HC.
18. O'Rourke et al. (2011)	767 pre‐HD 217 HC	Executive functions, processing speed, working memory	TMT, UHDRS, SDMT, Stroop test, WAIS‐III, Letter‐Number Sequencing, SCL‐90‐R, BDI–II	Behavioral	Comparisons between groups	In prodromal HD participants manifesting prodromal motor signs and psychiatric symptoms, the TMT primarily measures cognitive abilities. TMT scores differentiated between healthy comparisons and prodromal HD individuals as far as 9–15 years before estimated diagnosis.
19. Paz‐Rodríguez F. et al. (2024)	146 HD: 60 HDC (carriers) 86 non‐HDC (noncarriers)	Executive function	MMSE, SDMT, Stroop‐B, phonological fluency	Behavioral	Comparisons between groups	This study explores the cognitive performance of individuals at risk of developing HD, comparing those who are carriers of the huntingtin gene with those who are not. The results show that carriers close to the manifest stage have lower performance on tests assessing information processing speed and attention, suggesting that prefrontal cognitive dysfunction occurs years before the disease is diagnosed.
20. Reyes et al. (2021)	36 pre‐HD (12 M,45.08 ± 12.15) 28 HC (8 M, 43.96 ± 10.48)	Attention, verbal learning and memory, and planning and problem solving	UHDRS, STT, PST, SOT, The Neurocom Smart Balance Master, HADS, TMT, HVLT, The One Touch Stockings of Cambridge Test Pittsburgh Sleep Quality Index, Minnesota Leisure Time, Physical Activity Questionnaire, National Adult Reading Test, Cognitive Activity Scale, Australian Standard, Classification of Occupations, TMS	Behavioral	Comparisons between groups	Poorer sleep quality was associated with greater cognitive dual‐task cost in individuals with premanifest HD. Compared with HC, pre‐HD demonstrated an impaired capacity to dual task. Dual‐task measures exhibited acceptable test–retest reliability in premanifest HD and HC. These results show that dual‐tasking measures are sensitive and reliable in individuals with premanifest HD.
21. Robins Wahlin et al. (2010)	15 pre‐HD close (8 M, 45.00 ± 14.58) 15 pre‐HD distant (10 M, 49.29 ± 11.03) 34 noncarriers (13 M, 47.66 ± 9.66)	Executive function, language abilities, attention, abstract thinking, problem solving, visuospatial ability, intelligence	WAIS‐R	Behavioral	Comparisons between groups	The pre‐HD close group scored significantly lower on Verbal, Performance, and Full‐Scale IQ compared to noncarriers and performed significantly lower on 7 of the 11 WAIS‐R subtests, with low average scores in language abilities, attention, abstract thinking, problem solving, visuospatial ability, and psychomotor speed.
22. Snowden et al. (2002)	51 HD no mutation (29 M, 37 ± 11) 51 HD (18 M, 37 ± 9) 43 pre‐HD (24 M, 48 ± 13)	Memory, executive functions	WAIS‐R, Standardized Road Map Test of Directional Sense, object recall, story recall, word fluency, WCST, Picture sequencing test, Stroop test, Luria motor tasks	Behavioral	Longitudinal	Performance on psychomotor tasks in people with the mutation who were close to clinical onset of HD was intermediate between that of individuals many years from onset and those in the early stages of HD. In contrast, memory performance showed a more precipitous decline around the time of clinical onset of HD.
23. Stout et al. (2011)	738 pre‐HD: 202 near HD (84 M, 44.5 ± 9.8) 268 mid HD (102 M, 342.4 ± 9.9) 268 far HD (87 M, 37.0 ± 8.0) 168 HC (52 M, 43.9 ± 11.1)	Attention, working memory, psychomotor functions, episodic memory, language, recognition of facial emotion, sensory–perceptual functions, and executive functions	UHDRS, ANART, NART‐2, WAT, Stroop, SDMT, Tower 4 task, TMT, WAIS–III, WASI, Speeded tapping task, Benton Facial Recognition Test, Category learning task, Dynamic emotion recognition task, HVLT, Phonemic verbal fluency, Serial response time task, cued movement sequence task, self‐timed finger tapping task, University of Pennsylvania Smell Identification Test	Behavioral	Comparisons between groups: large‐scale study	The NEAR group performed significantly worse on most cognitive tests, while the MID group showed poorer performance on about half of them. Speeded finger tapping, self‐timed finger tapping, emotion recognition, and n‐back working memory tasks were particularly sensitive to prodromal HD effects, showing larger effect sizes near diagnosis.
24. Unmack Larsen et al. (2015)	48 HD (29 M; 51 ± 24.75) 50 pre‐HD (29 M; 36 ± 20.54) 39 controls (17 M; 40 ± 20.68)	Planning, decision making, monitoring, control of action, cognitive flexibility, set shifting, psychomotor speed, attention, memory, visuospatial functions, interference management, Internal attentional control, task analysis, strategy generation, regulation and response inhibition	DART, MMSE, MoCA, UHDRS, SDMT, Hamilton Depression Rating Scale (HAM‐D), Lexical alternating fluency and Semantic/lexical alternating fluency test, TMT B, Stroop interference test, Zoo map test, Brixton test, Hayling test	Behavioral	Comparisons between groups	In this study a distinction between presymptomatic and prodromal HD was observed across various cognitive areas, notably in motor speed, attention, executive function, verbal fluency, and memory. This reaffirms that disease progression correlates with a decline in cognitive abilities, particularly executive functions.
25. Verny et al. (2007)	44 Carriers of HD mutation (21 M; 32.7 ± 9.3) 39 noncarriers of HD mutation (15 M; 34.7 ± 9.0)	Global cognitive efficiency, psychomotor speed, attentional, executive and memory functions	Genetic counseling, Neurological consultation, Psychological interviews, MMSE, Mattis Dementia Rating Scale, Digit symbol subtest of the WAIS‐R, TMTA, Stroop test, Lexical fluency test, Category fluency test, Arithmetic subtest of the WAIS‐R, CVLT	Behavioral	Comparisons between groups	The study reveals that pre‐HD individuals, along with HD mutation carriers without clinical signs and HC, underwent neuropsychological evaluations focusing on cognitive efficiency, psychomotor speed, attention, executive functions, and memory. Pre‐HD subjects displayed notable variations in psychomotor speed, attention, and executive functioning tests prior to motor and cognitive alterations.
26. Wiecki et al. (2016)	122 pre‐HD (41 ± 8.7) 123 controls (46 ± 10) 125 HD (49.3 ± 9.8)	Executive control and response inhibition	Antisaccade task, UHDRS ‐ TMS, TFC	Behavioral	Comparisons between groups	The findings from this study indicate that executive control parameters can predict HD before the emergence of motor symptoms, whereas impairment in response inhibition processes occurs only after the onset of motor symptoms.

Abbreviations: BDI, Beck's Depression Inventory; CSSRS, Columbia Suicide Severity Rating Scale; CVLT, California Verbal Learning test; DART, Danish Adult Reading Test; GDT, Game of Dice Task; HADS, Hospital Anxiety and Depression Scale; HC, healthy controls; HD‐WPA, Huntington Disease‐ Word‐Problem Arithmetic; HD, Huntington Disease; HVLT, Hopkins Verbal Learning Test; IGT, Iowa Gambling Task; IQ, intelligence quotient; MMSE, Mini‐Mental State Examination; MoCA, Montreal Cognitive Assessment; NART, National Adult Reading Test; PBA, Problem Behaviors Assessment; PD‐CRS, Parkinson's disease‐cognitive rating scale; pre‐HD, presymptomatic HD; PST, dual‐task progressive subtraction test; QNE, Quadrature Null Effect; RT, reaction time; SCL‐90‐R, Symptom Check List‐90‐revised; SDMT, Symbol Digit Modalities Test; SOT, sensory organization test; STT, dual‐task serial threes test; TFC, Total Functional Capacity; TMS, total motor score; TMT, trail making test A and B; UHDRS, Unified Huntington Disease Rating Scale; WAIS‐R, Wechsler Adult Intelligence Scale‐Revised; WASI, Wechsler Abbreviated Scale of Intelligence.

**TABLE 2:** All studies related to the symptomatic phase of the disease.

References	Participants	Cognitive domains assessed	Endpoint	Type of measure	Methodological comments	Results
1. Allain et al. (2005)	10 HD (5 M, 38.8 ± 9.9) 12 HC (5 M, 41.5 ± 12.4)	Executive function	Arithmetic word‐problem‐solving task, MMSE, UHDRS, Mattis Dementia Rating Scale	Behavioral	Comparisons between groups	Patients with HD performed the solvable problems significantly worse than the HC but there was no difference in performance between the two groups in inhibiting aberrant problems.
2. Anderson et al. (2001)	27 HD (19 M, 49.8± 9.8)	Executive function	The Yale‐Brown Obsessive–Compulsive Scale, Verbal Fluency, SDMT, Stroop Color and Word Test, TFC	Behavioral	Comparisons between groups	Patients with obsessive or compulsive symptoms showed significantly greater impairment on neuropsychological tests measuring executive function than those without such symptoms.
3. Atkins K.J. et al. (2024)	17 HD (12 M; 53.3 ± 10.3) 22 HC (12 M; 50.9 ± 11.2)	Executive functions, motivation	UHDRS, MoCA, HVLT, SDMT, HADS, AES, DAS, RSVP, Physical effort task	Behavioral	Comparisons between groups	This study highlights the significant role of apathy in HD disease, revealing that individuals with HD show a notable aversion to cognitive effort compared to age‐matched controls. This suggests that their reduced motivation may stem from a motivational deficit rather than just the cognitive and physical challenges they face.
4. Bachoud‐Lévi et al. (2001)	22 HD (13 M, 42.0 ± 6.4)	executive functions, attention, language comprehension, visuospatial abilities, memory	The Token Test, the picture naming test, FCSRT, RAVLT, Mental Rotation Test, Judgment of Line Orientation Task, TFC, MADRS	Behavioral	Longitudinal study	Over the course of 4 years, this longitudinal study observed cognitive abilities in individuals with HD. The findings revealed a notable decline in cognitive function, particularly in attention and executive functions. Additionally, language comprehension and immediate visuospatial memory were also affected.
5. Beglinger et al. (2012)	74 HD (48.19 ± 12.32)	Executive functions phonemic verbal fluency psychomotor speed, attention, driving skills	UHDRS, TMS, The Controlled Oral Word Association Test, SDMT, Stroop Color and Word Test, RBANS, TMT, WAIS–III	Behavioral		In this study emerges that although driving status is associated with many aspects of the disease, results suggest that the strongest association is with cognitive performance, especially learning and psychomotor speed/attention. A detailed cognitive evaluation is an important component of multidisciplinary clinical assessment in patients with HD who are driving
6. Couette et al. (2008)	14 HD 14 HC	Spatial Attention	Cued response time	Behavioral	Comparisons between groups	HD patients’ attentional processing of visuo‐spatial stimuli may be slowed, with a tendency for attention to remain on peripherally cued locations. Also suggesting an alteration in the ability to disengage attention from a previous location and shift it to a new target.
7. Delval et al. (2008)	15 HD (43.9 ± 9.8) 15 HC (40.5 ± 10.5)	Executive function, attention	Dual‐task paradigm: gait test, counting backward	Behavioral	Comparisons between groups	Executive dysfunction and limited availability of attentional resources play a critical role in gait regulation in HD patients. The use of auditory cues during free gait and dual tasks did not improve kinematic parameters in HD patients, in contrast to the situation for HC subjects. HD patients have difficulty in synchronizing their footsteps with a metronome, mainly due to attentional deficits.
8. Devos et al. (2012)	30 HD (22 M, 50.20 ± 12.40) 30 HC (22 M, 50.26 ± 12.64)	Executive functions, attention, driving skills	UHDRS, TMT, MMSE, Letter Verbal Fluency Task, Stroop test, SDMT, driving simulation, FTDr	Behavioral	Cross‐sectional study	HD performed worse on all tests of the clinical battery and driving simulator than the HC. Fifteen patients with HD failed the FTDr evaluation. The SDMT, Stroop word reading, and TMTB provided the best model to predict FTDr, correctly classifying 26 patients.
9. Devos et al. (2014)	30 HD (22 M; 50.20 ± 12.40) 30 controls (22 M; 50.26 ± 12.64)	Information processing, executive control, selective attention, visual tracking and working memory. On‐road driving skills (skills of vehicle control, driving actions, attention, flexibility, adaptation strategies, adequate judgment, visuospatial, visuo‐ perceptual, and higher order cognitive skills)	TRIP, Choice reaction time test, UHDRS, UFOV, Divided attention, Copy test of Rey–Osterrieth, complex figure, Visual scanning, TMT A and B, Executive control, Incompatibility	Behavioral	Comparisons between groups	The HD group performed worse on all on‐road items. The challenges faced during on‐road driving tests and the high failure rates indicate the need for early monitoring of driving fitness in individuals with HD.
10. Eddy et al. (2015)	25 HD (15 M; 54.84 ± 6.82) 20 controls (11 M; 49.10 ± 13.05)	Phonological fluency, working memory, functional capacity, attention, reading speed, frequency and severity of common psychiatric symptoms	Verbal Fluency, DOT‐A, TMT, Stroop Task, DSST, Test of Irregular Word Reading Efficiency, TMS, TFC, PBA	Behavioral	Comparisons between groups	Patients with HD exhibited deficits on all timed neuropsychological tasks but not on measures of accuracy. Poorer functional capacity was related to cognitive deficits and more severe motor symptoms. Motor and psychiatric symptoms were also related to cognitive performance.
11. Fielding et al. (2006)	8 HD (50.87 ± 7.31) 10 HC (50.60 ± 10.25)	Attention	MMSE, BDI, The digit span task	Behavioral and SOA	Comparisons between groups	At the longest SOA, a clear differentiation between these groups was found for the intermediate SOAs. Unlike controls, where IOR manifested between 350 and 1000 ms, IOR was evident as early as 150 ms for HD patients.
12. Finke et al. (2006)	18 HD (5 M, 46.7 ± 10.6) 18 HC (5 M, 46.8 ± 10.9)	Visual attention, processing speed, visual working memory	Partial‐report paradigm	Behavioral	Comparisons between groups	In this study, a group of patients with advanced‐stage HD was evaluated using a TVA‐based whole‐ and partial‐report paradigm and compared with HC. The method provided estimates of parameters for spatial attention lateralization as well as two nonspatial aspects of attentional functions: processing speed and working memory capacity. Patients with HD were found to be impaired in all aspects. However, the spatial and nonspatial deficits were not correlated, indicating that they represent distinct attentional deficits with possibly different underlying neuropathological mechanisms.
13. Finke et al. (2007)	10 HD (7 M, 41.9 ± 8.6) 10 HC (6 M, 44.6 ± 11.3)	Attention, processing speed, working memory	MMSE, simultaneous‐perception task, whole‐report task	Behavioral	Comparisons between groups	The results demonstrate the presence of deficits in simultaneous perception in HD, related to a severe reduction in perceptual processing speed.
14. Galvez et al. (2017)	19 early HD (8 M; 45 ± 12) 19 HC (8 M; 45 ± 13)	Decision making	UHDRS, Cambridge gambling task	Behavioral	Comparisons between groups	This study evaluates whether the decision‐making process in patients is related to true risk‐taking behavior or to impulse control deficits. The results suggest the presence of impulse control deficits in HD gene carriers, but an inability to judge risk‐taking.
15. Georgiou‐Karistianis et al. (2012)	14 HD (11 M, 51.2 ± 10) 13 HC (9 M, 51 ± 9.3)	Attention	Attentional blink paradigm RSVP task	Behavioral	Comparisons between groups	In this study, an attentional blink paradigm was used to verify whether attentional control is impaired in HD. The results show that the progressive frontoparietal cortical alterations compromise the mechanisms of attentional control.
16. Hennig et al. (2014)	52 HD	Driving competency, speed of processing, working memory, executive function, semantic and phonemic verbal fluency, reaction time	RBANS, TMT, Stroop test, California Computerized Assessment Package, Grooved Pegboard Test.	Behavioral		The present study examines the usefulness of specific neuropsychological measures in predicting the actual driving competence in HD patients. Strong relationship between scores on a simple battery of four neuropsychological tests and driving competency.
17. Ho et al. (2003)	61 CAPIT‐HD short battery (40 M, 45.7 ± 9.0) 34 CAPIT‐HD baseline tests (24 M, 44.9 ± 8.1) 21 CANTAB‐HD battery (15 M, 47.2 ± 10.7)	Attention, executive function, memory	CAPIT‐HD, CANTAB	Behavioral	Longitudinal study	Patients showed a progressive impairment in attention, executive function, and immediate memory, with timed tests of psychomotor skill being particularly sensitive to decline. In contrast, general cognition, semantic memory, and delayed recall memory were relatively unaffected.
18. Holl et al. (2013)	18 early HD (53.56 ± 10.2) 20 controls (54.55 ± 13.1)	Risky decision making, inhibition of prepotent responses, internally guided word search and production, necessitating suppression of retrieval / production of inappropriate words and monitoring of the output	IGT, Stroop test, Verbal fluency test, MMSE, BDI, TFC, TMS	Behavioral	Comparisons between groups	Patients with early HD were significantly impaired on the Stroop and verbal fluency tests relative to controls. However, IGT performance was comparable across the 2 groups. This pattern of selective executive dysfunction in early HD probably reflects the fact that inhibitory processing involved in both the Stroop and verbal fluency tests recruits the dorsolateral caudate and its cortical connections, which are dysfunctional in early HD, whereas risky decision making during the IGT recruits the ventromedial caudate and its connections, which remain spared early on in the disease.
19. Hoth et al. (2007)	66 HD (39 M, 46.8 ± 10.0) 66 collaterals (22 M, 48.5 ± 10.7)	Executive function, decision making, attention, verbal fluency	The Patient Competency Rating Scale, UHDRS, BDI, WCST, IGT, Stroop Color–Word Test, SDMT, and the Letter Fluency Task	Behavioral	Comparisons between groups	Patients affected with HD demonstrated impaired awareness of their competency. Patients lacked awareness across symptom domains (behavioral control, emotional control, activities of daily living), which was significantly greater for their perception of their own behavior than for their perception of their collateral's behavior.
20. Jacobs et al. (2018a)	114 HD employed (54 M: 42.5 ± 10.5) 106 HD unemployed (42 M; 51.1 ± 10.0)	Executive functions (psychomotor speed, visual attention, speed of processing, cognitive flexibility)	UHDRS, SDMT, Stroop test, TMT, PBA	Behavioral	Group differences	HD with lower cognitive performances and higher apathy scores were more likely to be unemployed than were HD. Motor functioning was an independent predictor of unemployed and cognitive impairments were independent determinants of unemployed.
21. Johnson et al. (2017)	22 HD (13 M; 45.09 ± 12.68) 14 HC (4 M; 45.21 ± 14.31)	Executive functions (conflict resolution, attentional control, inhibitory control, orientation, attention/working memory, comprehension/praxis, naming, verbal recall/figure drawing)	BIS‐11, BIS/BAS, The Frontal Systems Behavior Scale, Flanker Task, MMSE	Behavioral	Comparisons between groups	The study reveals that trait impulsivity is reported to be higher in HD and may not be driven by an altered evaluation of reward and the appetitive nature of stimuli, but rather by an increased executive dysfunction and lack of sensitivity to punishment.
22. Julayanont et al. (2020)	307 HD: 31 HD‐Norm (54.39 ± 9.59) 144 HD‐MCI (48.49 ± 11.98) 132 HD‐Dem (55.10 ± 11.65)	Executive functions, processing speed, language, memory, visuospatial attention, visuospatial working memory	The letter‐phonemic fluency task, TMT A and B, The Stroop Interference Test, HVLT‐R, Animal fluency test, UHDRS	Behavioral	Longitudinal	A study found that patients with HD often develop Mild Cognitive Impairment after 5 years of motor symptoms, with early deficits in executive function, processing speed, language, and memory, indicating that cognitive decline affects multiple domains in these patients.
23. Klempíř et al. (2009)	45 HD	Short‐term memory, executive functions	The Stroop test, TMT, SDMT, verbal fluency test, AVLT	Behavioral		Voluntary components of motor performance were found to be significantly correlated with verbal short‐term memory disturbances, with tests of executive functions more dependent on motor performance and also with tests of executive functions less dependent on motor performance.
24. Kloos et al. (2017)	70 HD (50.9 ± 14.6)	Attention, perceptual speed, visual scanning and linguistic functions	UHDRS, Tinetti Mobility Test	Behavioral	Longitudinal study	Patients with Huntington's disease who experience cognitive deficits also face difficulties with walking and balance. They found significant correlations between executive function measures and those of walking, balance, and mobility.
25. Lawrence et al. (1996)	18 HD (12 M, 44.1 ± 12.2) 18 HC (41.9 ± 16.3) 30 HC2 (42.4 ± 19.9)	Executive functions, memory	Kendrick Object Learning Test, MMSE, CANTAB, Corsi block‐tapping task, spatial recognition task, task of discrimination learning, spatial working memory	Behavioral	Comparisons between groups	Patients with early HD were found to have a wide range of cognitive impairments encompassing both visuospatial memory and executive functions. In contrast to patients with more advanced HD, early HD patients were not impaired at simple reversal learning, but were impaired at performing an extradimensional shift.
26. Lawrence et al. (1998)	22 carriers (9 M, 39.4 ± 10.1) 32 noncarriers (13 M, 41.4 ± 9.6)	Executive function, attention, memory	CANTAB, NART, WAIS‐R, Spatial span, task of spatial working memory, One‐touch ToL	Behavioral	Comparisons between groups Double‐blind	HD mutation carriers performed significantly less well than noncarriers, matched for age and IQ, on tests of attentional set shifting and semantic verbal fluency. Furthermore, performance on these two tests was significantly correlated, even after partialling out the effects of age and IQ.
27. Lawrence et al. (2000)	19 HDa (47.5 ± 9.8) 19 HDb (42.7 ± 11.3) 21 HDc (41.8 ± 9.2) 20 HCa (43.4 ± 16.7) 20 HCb (43.4 ± 15.1) 17 HCc (37.7 ± 15.3)	Attention, working memory, visuospatial cognition	MMSE, UHDRS, PAL, NART, DMTS, VSMTS, CANTAB, VOSP	Behavioral	Comparisons between groups	In this study HD exhibited deficits on tests of pattern and spatial recognition memory, showed impaired simultaneous matching and delay independent delayed matching to sample deficits, showed spared accuracy but impaired reaction times in visual search, were impaired in spatial but not visual object working memory, and showed impaired pattern location associative learning.
28. Lemiere et al. (2004)	19 HD (12 M, 49.91 ± 12.21) 12 AC (7 M, 39.36 ± 10.89) 11HC (3 M, 40.61 ± 4.37)	Intelligence, attention, memory, language, visuospatial perception, executive functions	SDMT, Stroop Color and Word, Boston Naming Test, Object and Space Perception, TMT‐B, Block Span, Digit Span Backward, HVLT, Conditional Associative Learning Test	Behavioral	A longitudinal follow‐up study	The study shows that the tasks measuring mainly attention, object and space perception and executive functions adequately assess the progression of HD disease. Other cognitive functions do not significantly deteriorate.
29. McLauchlan et al. (2019)	51 HD (26 M) 26 HC (17 M)	Executive functions, verbal fluency	AES, PBA, BISBAS, TMS, Phonemic Verbal Fluency, Balloon Analogue Risk Task, RRTT, Persistence task	Behavioral	Comparisons between groups	Huntington's disease participants had deficits in instrumental learning with impaired response to loss, but no evidence to suggest altered reward‐related behavior or effort. An executive dysfunction contribution to apathy in HD.
30. Mörkl et al. (2016)	29 Late‐stage HD (17 M; 49.21 ± 11.13) 23 Early‐stage HD (15 M; 45.78 ± 11.23) 34 controls (19 M; 49.06 ± 17.38)	Executive functions: mental problem solving, planning, behavioral inhibition and impulse control	TMS, Mehrfachwahl–Wortschatz–Intelligenz test, ToL	Behavioral	Comparisons between groups	Early‐ and late‐stage Huntington's disease (HD) pathology impacts executive subdomains differently. Accuracy varies between early‐ and late‐stage HD patients, but other areas like planning time and number of breaks do not.
31. Purcell et al. (2020)	17 HD (10 M,55 ± 9.7) 17 HC (9 M,56.5 ± 9.3)	Attention, working memory, information processing speed, visuospatial perception, verbal fluency	Two‐minute walk test with APDM: Self‐Selected, Fast‐as‐Possible Dual task: Verbal fluency task, Animal naming task TMS, MoCA, Digit Span forward, backward, and sequencing (WAIS‐IV), SDMT, CERAD Word List Memory, delayed recall portion, Judgment of Line Orientation, The Berg Balance Scale (BBS), Activities‐Specific Balance Confidence Scale (ABC)	Behavioral	Comparisons between groups	In high‐speed walking tests combined with cognitive tasks, individuals with Huntington's disease (HD) exhibited difficulties in walking and turning movements. Specifically, HD patients walked slower and took shorter strides compared to healthy controls (HC). Additionally, during dual‐task scenarios involving rotations, HD patients experienced greater challenges, needing more time and steps to complete turns than the control group.
32. Reedeker et al. (2010)	96 HD (45 M, 51 ± 11)	Semantic memory, attention, working memory, visuoverbal speed	UHDRS, CIDI, MMSE, Apathy Scale, Verbal Fluency test, SDMT, Stroop, TFC	Behavioral	Comparisons between groups: cross‐sectional	The results of this study suggest that hypokinesia may have a significant impact on the global and cognitive functioning of HD patients. The presence of moderate to severe hypokinesia in HD patients co‐occurs with executive cognitive dysfunction and adversely affects global functioning.
33. Rodrigues et al. (2009)	41 HD (15 M, 48.8 ± 13.6) 53 HC (15 M, 48.25 ± 13.8)	Executive function, orientation, language, memory, visual ability, attention	UHDRS, FAB, Verbal Fluency Test, SDMT, Stroop Interference Test, MMSE	Behavioral	Comparisons between groups	The findings indicate that the FAB could be useful in distinguishing HD patients from HC and for monitoring cognitive deterioration over time. The 1‐year longitudinal evaluation revealed a global trend toward a worsening in the second score of the FAB (mental flexibility).
34. Rothlind et al. (1993)	80 HD (40 M, 46.1 ± 12.1)	Attention, memory, intelligence	HD‐ADL, MMSE, digit span, CERAD word list learning test, the controlled oral word association test, go/no‐go test, Stroop color, Stroop word test, Stroop color words, TMT A and B, Benton visual retention test, Luria hand position sequencing test, WCST, QNE	Behavioral		The results demonstrate a moderately high correlation between impairment in everyday functioning and performance on neuropsychological tests and measures of control motor. Findings suggest that psychomotor speed and the ability to regulate attention may be particularly important determinants of everyday functioning in mild HD.
35. Snowden et al. (2001)	87 HD (51 M, 45 ± 12) 55 HC (21 M, 42 ± 9)	Executive function, memory	WAIS, Digit span forward and backward, Standardized Road Map Test of Directional Sense, object recall, story recall, category fluency, letter fluency, WCST, Picture Sequencing Test, Stroop test	Behavioral	Longitudinal	These findings imply that various neuropsychological tests may be sensitive to distinct types of cognitive changes in HD patients as time progresses. The data highlight the slow progression of HD, the limitations of standard cognitive tests in detecting change over short periods, and the need for therapeutic studies that encompass a relatively prolonged time frame.
36. Solca et al. (2015)	24 HD (52.33 ± 13.82) 24 Controls (52.46 ± 14.02)	Counter factual thinking (CFT): ability of learning from past experience and predicting future events. Logical‐abstract reasoning, short‐ and long‐term verbal memory, verbal comprehension, frontal, attentive and executive functioning	UHDRS, Spontaneous Counterfactual Generation Test, CIT, Mini‐Mental State Examination (MMSE),Raven Progressive Matrices Test, Rey's Words Test, Token Test, FAB, TMT, SDMT, Stroop Interference Test, BDI, CIT, STAI‐Y, SCL‐90, SF‐36, DEX‐S, SES, BFQ, Phonemic Verbal Fluency Test, Rotter Internal‐External Locus of Control Scale	Behavioral	Comparisons between groups	The results show a significant impairment in the spontaneous generation of CFT and low performance on the Counterfactual Inference Test (CIT) in HD patients. Low performance on the spontaneous CFT test significantly correlates with impaired attention abilities, verbal fluency and frontal lobe efficiency, as measured by TMT– Part A, Phonemic Verbal Fluency Test and FAB.
37. Solca et al. (2022)	38 HD (18 M, 60.18 ± 9.85) 73 HC (24 M, 55.97 ± 14.45)	Executive function, memory, language, visuospatial skills and orientation	MoCA, UHDRS, FAB	Behavioral	Comparisons between groups	The results of the study indicate that the FAB has demonstrated optimal diagnostic properties. The study provides evidence on the usefulness of the FAB as a cognitive screening tool in Huntington's disease and the importance of assessing cognitive functions.
38. Sprengelmeyer et al. (1995)	20 HD (15 M, 42.0 ± 11.8) 27 HC (15 M, 38.1 ± 11.9)	Executive functions, attention	Computerized test‐battery for attention	Behavioral	Comparisons between groups	The study's results show that HD patients have difficulties with several attentional tasks, such as maintaining vigilance, divided attention, response flexibility, and response inhibition.
39. Thompson et al. (2010)	14 HD (2 M; 49 ± 10) 14 controls (4 M; 49 ± 12)	Attention, capacity to automatize behavior and cognitive timing functions	UHDRS, MMSE, Finger‐tapping task (single task and dual task)	Behavioral	Comparisons between groups	HD individuals struggle to automate behaviors, which may contribute to their difficulty in performing multiple tasks at the same time.
40. Tsai et al. (1995)	25 HD1 16 HD2 24 HC	Visual attention	Reaction time, MMSE, QNE	Electro‐oculography	Comparisons between groups	HD patients face more challenges in directing visual attention compared to HC, demonstrated by delays in saccade latency and thumb reaction times. The ability to direct visual attention is normal in HD and are compatible with the hypothesis that in HD, frontal–basal ganglia circuits are more affected than parietal lobe pathways.
41. Van Liew et al. (2013)	31 HD (53.35 ± 11.33) 32 gene‐ positive AR (42.31 ± 10.89)	Cognitive functions	UHDRS, Social Problem‐Solving Inventory‐Revised, SCL‐90, Stroop Color, Word, and Interference, SDMT, MMSE, MoCA	Behavioral		Cognitive and motor states uniquely and significantly predicted function in HD; however, neither psychiatric nor SPS states did. SPS was, however, significantly related to motor, cognitive, and psychiatric states, suggesting that it may bridge the correlative gap between psychiatric and cognitive states in HD.
42. Van Wouwe et al. (2016)	18 HD (7 M; 48.7 ± 12.6) 17 controls (5 M; 45.88 ± 12.8)	Decision making and risk taking	Cohen's risk‐taking task (adapted)	Behavioral	Comparisons between groups	Concerning significant implications for everyday decision making, such as financial choices, HD patients display a tendency toward opting for high‐risk decisions, even when fully aware of the long‐term consequences. This behavior might be linked to specific changes in how they assess rewards.
43. Vaportzis et al. (2015a)	15 HD (10 M; 58.40 ± 8.87) 15 HC (10 M; 55.53 ± 12.33)	Divided attention, proprioception, attention allocation	Dual tasks: circle tracing and serial subtraction. UHDRS, MoCA, WAIS, Inventory of Depressive Symptomatology‐Self‐report	Behavioral	Comparisons between groups	HD participants were significantly slower and less accurate than HC. Both groups were slower and less accurate when performing indirect circle tracing compared with direct circle tracing. HD experienced greater dual‐task interference in terms of accuracy when performing direct circle tracing compared with indirect circle tracing. HC were more inclined to speed– accuracy trade‐offs compared with HD.
44. Vaportzis et al. (2015b)	14 HD (10 M; 57.86 ± 8.94) 14 controls (10 M; 58.64 ± 11.39)	Attention and dual tasking	MoCA, Wechsler Test of Adult Reading, Inventory of Depressive Symptomatology‐Self‐Report, TFC, Oculomotor score, Cancellation task	Behavioral	Comparisons between groups	The findings indicated that individuals with HD did not exhibit slower or less accurate performance compared to the control group. However, they did experience more interference when performing dual tasks, particularly in terms of speed.
45. Watkins et al. (2000)	20 HD (12 M, 46.7 ± 10.2) 20 HC (9 M, 44.4 ± 13.2)	Executive function, decision making, visuo‐spatial planning	ToL, Stroop, WAIS, BDI, NART, MMSE, CANTAB	Behavioral	Comparisons between groups	HD were significantly less accurate than HC on the planning test. On the decision‐making task, HC were unimpaired on the quality of their decision making.
46. Wibawa et al. (2020)	28 HD no anosognosia 10 HD anosognosia	Executive functions	ZBI, CBI, UHDRS, MMSE, Stroop test, TMT, Verbal Fluency test, SDMT	Behavioral	Comparisons between groups	Patients with anosognosia elicited higher caregiver burden ratings on both the ZBI and CBI while also demonstrating poorer executive function. Except for CAG burden score, between‐group characteristics did not differ significantly. Stroop Interference predicted both anosognosia and caregiver burden.

Abbreviations: ABC, Activities‐Specific Balance Confidence Scale; AES, Apathy Evaluation Scale; AVLT, Rey Auditory Verbal Learning Test; BBS, The Berg Balance Scale; BDI, Beck Depression Inventory; BFQ, Big‐Five Questionnaire; BIS‐11, Barratt Impulsivity Scale; BIS/BAS, Inhibition/Behavioral Activation Scale; BISBAS, Behavioral Inhibition, Behavioral Activation Scale; CANTAB, Cambridge Neuropsychological Test Automated Battery; CAPIT‐HD, Core Assessment Protocol for Intrastriatal Transplantation; CBI, Caregiver Burden Inventory; CIDI, Cognitive Impairment Diagnosing Instrument; CIT, Counterfactual Inference Test; DAS, Depression Anxiety and Stress Scale; DEX‐S, Dysexecutive Questionnaire Subject Form; DMTS, Delayed Matching Condition; DOT‐A, Digit Ordering Test‐Adapted; DSST, Digit Symbol Substitution Test; FAB, Frontal Assessment Battery; FCSRT, Free and Cued Selective Reminding Test; FTDr, fitness to drive; HADS, Hospital Anxiety and Depression Scale; HC, healthy controls; HD, Huntington Disease; HVLT, Hopkins Verbal Learning Test; IGT, Iowa Gambling Task; IOR, Inhibition of response; MADRS, Montgomery–Asberg Depression Rating Scale; MMSE, Mini‐Mental State Examination; MoCA, Montreal Cognitive Assessment; NART, National Adult Reading Test; PAL, Paired Associates Learning Task; PBA, Problem Behaviors Assessment; pre‐HD, presymptomatic HD; QNE, Quantified Neurological Examination; RAVLT, Rey Auditory Verbal Learning Test; RBANS, Repeatable Battery for the Assessment of Neuropsychological Status; RSVP, Rapid serial visual presentation; SCL‐90, Symptoms Check List; SDMT, Symbol Digit Modalities Test; SES, Rosenberg Self Esteem Scale; SF‐36, Short Form‐36 Health Survey; SOA, stimulus onset asynchronies; STAI‐Y, State Trait Anxiety Inventory; TFC, Total Functional Capacity; TMS, total motor score; TMT, trail making test; TRIP, Test Ride for Investigating Practical fitness to drive; TVA, theory of visual attention; UFOV, Useful Field of View; UHDRS, Unified Huntington's Disease Rating Scale; VOSP, Visual Object and Space Perception Battery; VSMTS, Visual Search Matching To Sample; WAIS, Wechsler Adult Intelligence Scale; WCST, Wisconsin Card Sorting Test; ZBI, Zarit Burden Interview.

**TABLE 3:** All studies related to neurophysiological and neuroimaging methods used to assess executive dysfunction.

References	Participants	Cognitive domains assessed	Endpoint	Type of measure	Methodological comments	Results
1. Aron et al. (2003)	18 HD (12 M, 47.7 ± 8.9) 18 PFC left (11 M, 51.9 ± 10.0) 19 PFC right (12 M, 53.6 ± 10.0) 19 HC (10 M, 48.1 ± 9.4)	Selective attention	NART, reaction time	Behavioral and structural (MRI)	Comparisons between groups	HD and frontal groups were significantly more distractible than HC for RT, but they had a different pattern of errors. It is argued that a reactive‐inhibition mechanism, required in the circumstance of strong distractor activation, is affected by frontal damage, while a lateral‐inhibition mechanism, invoked during the recruitment of selective attention, is affected in HD.
2. Beste et al. (2015)	Baseline: ‐ 15 EBL‐ preHD (6 M; 40.4 ± 10.5) ‐ 14 NoEBL‐ preHD (7 M; 36.93 ± 10.7) 21‐month: ‐ 14 EBL‐ preHD (6 M; 42.1 ± 10.2) ‐ 12 NoEBL‐ preHD (6 M; 38.63 ± 11.1)	Attentional selection	TISL, Attentional selection paradigm, EEG, ERPs UHDRS	Behavioral and electrophysiological (EEG, ERPs)	Longitudinal study	The results show that attentional selection processes decline during premanifest disease progression. Importantly, the efficacy of protocols used to induce neural plasticity in processes underlying attentional selection processes also increases in course of ongoing neurodegeneration in premanifest HD. This was reflected in behavioral data and electrophysiological correlates of processes related to the allocation of attention.
3. Beste et al. (2007)	11 pre‐HD (33.70 ± 9.69) 9 HC (34.00 ± 9.89)	Memory, attention	Flanker task, TFC, IS, BDI, MMSE, WAIS, Digit span, Benton test, VLMT	Behavioral and electrophysiological (ERP)	Comparisons between groups	No parameter of the behavioral data differed between the groups. A selective increase in the power of the cognitive delta‐Ne component was found in pre‐HD relative to controls.
4. Beste et al. (2008a)	11 HD (6 M, 39.81 ± 8.96) 14 pre‐HD (7 M, 35.91 ± 10.03) 12 HC (6 M, 36.50 ± 8.64)	Attention	Flanker task, MMSE, BDI, YMRS	Behavioral and electrophysiological (ERP)	Comparisons between groups	In the behavioral data, we found a general response slowing in HD. The ERP data show a decrease of the N1 on the flanker in HD and pre‐HD; this suggests deficient attentional processes. LRP was unchanged, whereas the late LRP was delayed in HD. The N2 was reduced in the HD but not in the pre‐HD and the control groups.
5. Beste et al. (2008b)	13 HD (37.6 ± 9.5) 13 pre‐HD (37.07 ± 9.1) 12 HC (37.9 ± 10.1)	Executive function, memory, attention	Stroop test, WAIS, Benton, WMS‐R, AVLT, MMSE, BDI, YMRS	Behavioral and electrophysiological (ERP)	Comparisons between groups	Selectively enhanced cognitive functioning can emerge together with otherwise impaired cognitive functioning. Increased activity of the NMDA‐receptor system, might facilitate signal propagation at striatal level that enables more efficient task execution through a winner‐take‐all process.
6. Beste et al. (2011)	25 pre‐HD (11 M, 39.36 ± 10.04) 25 HC (11 M, 39.12 ± 9.5)	Response inhibition	UHDRS, Go/Nogo task	Behavioral and electrophysiological (EEG)	Comparisons between groups	Pre‐HDs only encounter problems in response inhibition, when discordant contextual information and sensory input have to be integrated. Efficient controlled processes of action seem to be closely dependent upon highly reliable neural synchronization processes.
7. Beste et al. (2012)	30 pre‐HD (14 M, 38.66 ± 10.54) 30 HC (14 M, 39.55 ± 8.9)	Working memory, flexible response adaptation, control of conflicting information	UHDRS, Stroop	Behavioral and electrophysiological structural (EEG, MRI)	Comparisons between groups	The results show that pre‐HD revealed higher switch costs when task switching and the resolution of conflict occurred in parallel (switch incompatible). The N2 group differences (attenuate in pre‐HD) were only evident on incompatible switch trials. Pre‐HD group showed prolongation of P3 latency, reductions of P3 amplitude and evoked wavelet power respect to HC; this pattern reflected increased working memory load or modulations in attentional set shifting.
8. Casella et al. (2021)	19 HD (12 M,41.61 ± 13.1) 21 HC (10 M, 45.14 ± 12.5)	Executive function, verbal working‐memory, spatial working‐memory, social cognition, motor speed	MRI, MoCA, TOPF‐UK, N‐back, Digit span test from the WAIS‐R, Visual patterns test, Eyes test, Finger tapping task	Behavioral and functional (fMRI)	Comparisons between groups	In HD patients, the myelin water signal fraction (fm) was lower, but there were no differences in myelin and intra‐axonal water pools (Δω) between groups. Tests showed that HD patients experienced a greater age‐related decline in executive function, confirming earlier findings of executive dysfunction in HD. There was a positive correlation between executive function and fm, which was also examined in relation to how close patients were to the onset of the disease.
9. Cheng et al. (2014)	8 HD (6 M; 45.9 ± 2.4) 8 controls (5 M; 47.5 ± 2.5)	Involuntary attention processing	Magnetic mismatch responses, UHDRS	Magnetophysiological	Comparisons between groups	The study found a reduced automatic frontal response to auditory deviance in HD patients. This reduction was revealed by MMNm analysis. Huntington's disease patients showed a prolonged and reduced MMNm response in the left hemisphere compared to the control group. Furthermore, a significant reduction in MMNm strength was observed in bilateral frontal responses in Huntington's disease patients. These results suggest a decline in information processing speed in symptomatic HD patients.
10. Domínguez D et al. (2017)	35 pre‐HD (41.7 ± 9.8) 18 HD (50.4 ± 7.2) 29 controls (42.4 ± 12.3)	Set‐shifting abilities Executive functions	Shifting response set task Frontal Systems Behavior Scale Schedule of Obsessions, Compulsions and Pathological Impulses	Behavioral and functional (fMRI)	Comparisons between groups	Over 30 months, no longitudinal BOLD changes were detected in pre‐HD) compared to controls. In contrast, HD patients showed greater BOLD decline in task‐related (anterior cingulate, striatum) and default mode regions (medial prefrontal, posterior cingulate/precuneus). Across stages, reduced BOLD in the right dorsolateral prefrontal cortex and putamen related to executive and behavioral deficits, and broader frontostriatal and default mode reductions tracked disease severity. These results suggest that progressive network changes underlie functional decline in HD and may inform targeted interventions to preserve cognition and reduce psychiatric symptoms.
11. Ferraro et al. (2014)	16 preHD (8 M; 31 ± 7.9) 18 controls (9 M; 29 ± 5.4)	Global cognitive abilities, attention, executive functions, visual spatial attention	Milan overall dementia assessment, Raven's progressive matrices, forward and backward digit span, Dual Task, Stroop‐Color‐Word, Interference Task, Phonemic Fluency, Ruff figural fluency test, TMT, attention matrices, fMRI	Behavioral and functional (fMRI)	Comparisons between groups	The study shows that changes in BOLD signals in the frontal cortex could serve as a potential biomarker. Specifically, a network of brain activity involving the oculomotor frontal cortex has been identified, which appears to be sensitive to early pathological changes.
12. Harrington et al. (2014)	325 preHD (40.7 ± 10.2) 119 controls (42.4 ± 11.4)	Attention and information processing speed, working memory, verbal learning and memory, negative emotion recognition and temporal processing	UHDRS, SDMT, WAIS‐III Letter‐Number Sequencing, HVLT‐R, Paced timing task, Computerized emotion recognition task	Behavioral and imaging (MRI)	Comparisons between groups	The results showed that performances in different cognitive domains that are vulnerable to decline in pre‐HD were correlated with regionally specific patterns of cortical and striatal morphometry. Putamen and/or caudate volumes were top‐ranked correlates of performance across all cognitive domains, as was cortical thickness in regions related to the processing demands of each domain.
13. Harrington et al. (2015)	16 LowHD (1 M; 32.6 ± 9.0) 16 MediumHD (5 M; 39.3 ± 9.7) 16 HighHD (2 M; 47.1 ± 12.6) 16 controls (4 M; 42.6 ± 9.2)	Executive functions; selective attention, cognitive flexibility	Stroop Color and Word Test, SDMT, TMT	Behavioral and functional (fMRI)	Comparisons between groups	In individuals in the prodromal phase of HD, abnormalities in brain connectivity have been linked to impairments in executive functions. Specifically, disrupted connectivity in the right inferior parietal gyrus, the right thalamus, and the left anterior cingulate cortex has been associated with poorer performance on executive function tests such as the Stroop test and the TMT.
14. Hart et al. (2012)	12 pre‐HD (6 M, 43 ± 10) 17 HD (8 M, 50 ± 11) 15 HC (7 M, 51 ± 10)	Attention, inhibition	UHDRS, SART	Behavioral and electrophysiological (EEG‐P300)	Comparisons between groups	The HD made more Go errors and reacted slower than the other groups. P300 data showed that for HD the No‐go amplitude was lower than for the other two groups. HD showed a reduced capacity to effectively control attention. They proved unable to resume the task directly after having made an error, and need more time to return to pre‐error performance levels. No attentional control deficits were found for the pre‐HD.
15. Jurgens et al. (2011)	14 pre‐HD 12 noncarriers	Attention	UHDRS, The Sustained Attention to Response Task	Behavioral, electrophysiological (EEG) and structural (MRI)	Comparisons between groups	P3 amplitude and latency did not differ between groups. In carriers, longer P3 latency during Go‐trials was strongly associated with smaller caudate, putamen and globus pallidus volumes The exceptionally strong relations of P3 latency with basal ganglia volumes in carriers suggest that the P3 may provide a marker for disease progression in HD.
16. Kamble et al. (2018)	32 HD (20 M; 42.1 ± 14.1) 30 controls (39.4 ± 12.4)	Attention, verbal fluency, executive function, visuospatial function, learning and memory	UHDRS, MMSE, FAB, MoCA, Digit span forward and backward, Verbal fluency test, Corsi block‐tapping test, Rey's AVLT, Story recall test	Behavioral and transcranial magnetic stimulation (TMSs)	Comparisons between groups.	A significant impairment in attention, verbal fluency, executive function, visuospatial function, learning, and memory was observed in HD patients. There was no correlation between cortical excitability changes and cognitive impairment.
17. Kassubek et al. (2005)	44 HD (21 M, 44.7 ± 10.7) 22 HC (10 M, 44.1 ± 16.9)	Executive functions, attention	Stroop color word, Digit Symbol, WAIS‐R	Behavioral and structural (MRI)	Comparisons between groups	Besides striatal areas, symmetrical regional atrophy of the thalamus was found to covary significantly with cognitive performance. These results suggest that thalamic degeneration contributes in an important way to the impairment of executive function in early HD. Patients who are impaired in executive tests display structural double lesions of the basal‐ganglia‐thalamo‐cortical circuitry both at the striatal and at the thalamic level.
18. Kim et al. (2017)	14 pre‐HD (6 M; 41.8 ± 13.2) 11 controls (5 M; 46.2 ± 11.9)	Executive function, spatial and verbal working memory, cognitive control and fluency	NIH EXAMINER	Behavioral imaging (MRI)	Comparisons between groups	From this study, it emerges that pre‐HD patients exhibit a reduction in the bilateral global volume of the caudate nucleus compared to HC. This decrease in caudate volume is associated with early structural damage and may be related to cognitive deficits, particularly in executive function and working memory.
19. Koenig et al. (2014)	48 pre‐HD 32 HC	Cognitive flexibility, cognitive inhibition, attention, processing speed	UHDRS, TMT, Stroop Color‐Word Interference task, SDMT	Behavioral and functional (fMRI)	Comparisons between groups	This study found that changes in functional connectivity within motor areas and long‐range connections are associated with poorer performance on cognitive‐motor tasks. These connectivity alterations might contribute to deficits in executive control of movement as individuals approach a clear diagnosis of HD. Additionally, changes in interhemispheric interactions between sensorimotor areas could play a role in the motor symptoms of HD.
20. Langley et al. (2021)	51 pre‐HD (29.22 ± 5.71) 53 HC (28.85 ± 5.50)	Cognitive flexibility, visual discrimination learning, attentional set shifting	CANTAB, IED, NART, UHDRS	Behavioral and imaging (MRI)	Comparisons between groups	This study shows that cognitive flexibility is impaired in pre‐HD subjects. It also reveals that in HC, cognitive flexibility is linked to functional connectivity between the ventrolateral PFC and the ventral striatum. However, in pre‐HD participants, different frontostriatal circuits are involved in shifting attention, suggesting possible functional reorganization.
21. Martinez‐Horta et al. (2020)	35 HD (12 M, 51.8 ± 12) ‐HD with (HD‐Dem) and without (HDND) dementia 15 HC (11 M, 45.9 ± 8)	Attention, working memory, verbal fluency, executive function	MMSE, UHDRS, SDMT, TMT	Behavioral and imaging (MRI)	Cross‐sectional study Comparisons between groups	The findings of this study demonstrate that in HD, severe cognitive decline is associated with a broad pattern of cortical thinning and brain atrophy across cortical and subcortical regions, notably affecting parieto‐temporal and occipital areas. These areas are particularly prominent in distinguishing patients with severe cognitive impairment from those with normal or mild impairment.
22. Matsui et al. (2014)	53 preHD: ‐Low preHD (3 M; 32.1 ± 8.8) ‐Medium preHD (6 M; 39.4 ± 10.8) ‐High preHD (2 M; 47.8 ± 12.2) 34 controls (11 M; 49.1 ± 10.4)	Processing speed, executive functions, working memory	DWI, UHDRS, SDMT, Stroop Color Word Test, TMT	Behavioral and imaging (MRI)	Comparisons between groups	The authors assessed white matter integrity in patients with HD. They examined changes in fiber distribution within the dorsolateral prefrontal cortex and the orbitofrontal cortex in HD patients compared to HC. The findings revealed reduced white matter integrity in these regions. Additionally, the significance of these changes was highlighted by the correlation between white matter measurements and scores from a cognitive assessment known for detecting cognitive impairments in the prodromal stage of HD.
23. Matsui et al. (2015)	43 LowHD (9 M; 34.4 ± 8.6) 54 MediumHD (16 M; 40.8 ± 9.9) 49 HighHD (13 M; 45.3 ± 12.0) 65 controls (22 M; 46.4 ± 11.4)	Psychomotor speed, cognitive flexibility, working memory, processing speed and executive function	UHDRS, SDMT, TMT, Stroop Color and Word Test	Behavioral and imaging (MRI)	Comparisons between groups	The study reveals that HD leads to degeneration of white matter in the prefrontal cortex, detectable even in the early prodromal stages. These white matter changes are closely related to neuropsychological measures of executive function. This implies that the cognitive difficulties often observed in the prodromal phase of HD may be connected to these early white matter alterations.
24. Odish et al. (2018)	26 HD (10 M; 49.7 ± 8.5) 25 controls (7 M; 52.7 ± 8.7)	Attention, processing speed, functional capacity, working memory and visuospatial processing	qEEG, TMS, TFC, SDMT, Stroop Word Reading, BDI,‐II	Behavioral electrophysiological (EEG)	Comparisons between groups	qEEG analysis on subsets of electrophysiological features resulted in two highly significant correlations with clinical scores. The results of this pilot study suggest that qEEG may serve as a biomarker in HD. The indices correlating with modalities changing with the progression of the disease may lead to tools based on qEEG that help monitor efficacy in intervention studies.
25. Papp et al. (2013)	781 HD: ‐ 197 Low (35.09 ± 7.49) ‐ 278 Med (40.96 ± 9.46) ‐ 306 High (44.05 ± 9.81) 212 controls (43.65 ± 11.27)	Problem solving, strategy development, working memory, planning and insight	Towers Task, FrSBe, BDI‐II	Behavioral imaging (MRI)	Longitudinal study Comparisons between groups	Participants with more advanced HD showed slower and less accurate performance on the Towers Task, which was related to lower striatal and frontal white matter volumes. However, disease progression did not predict changes in performance over 4 years. While the Towers Task can measure executive dysfunction at baseline, it has limited sensitivity to detect cognitive decline over time in prodromal HD.
26. Pavese et al. (2010)	11 pre‐HD (5 M, 42.2 ± 7.8) 16 HD (11 M, 48.3 ± 6.1)	Executive function, attention	UHDRS	Behavioral and functional (PET)	Comparisons between groups	Symptomatic HD subjects with decreased cortical D_2_ dopamine receptor had worse scores on neuropsychological tests assessing attention and executive functions than subjects without cortical dopamine dysfunction. Results indicate that cortical dopaminergic dysfunction is common in both symptomatic and premanifest HD gene carriers.
27. Peinemann et al. (2005)	25 HD (13 M, 43.8 ± 7.7) 25 HC (13 M, 42.9 ± 9.8)	Executive function, planning ability, selective attention, response inhibition	Tower of Hanoi, Stroop Color Word Interference Test, WCST	Behavioral and structural 3D‐MRI	Comparisons between groups	Striatal atrophy in HD patients in early stages plays an important role not only in impaired motor control but also in executive dysfunction. Extrastriatal cortical areas seem to be involved in executive dysfunction as assessed by neuropsychological tests requiring for planning and problem solving, stimulus response selectivity and concept formation.
28. Possin et al. (2017)	Full sample: ‐ 16 preHD (44.9 ± 12.0) ‐ 17 controls (44.2 ± 15.9) MRI Subsample: ‐ 14 preHD (45.8 ± 11.9) ‐ 10 controls (41.9 ± 15.1)	Mental representation of spatial locations relative to landmarks (allocentric), and spatial locations relative to the viewer (egocentric)	UHDRS, MoCA, Egocentric and Allocentric Working Memory Tests	Behavioral imaging (MRI)	Comparisons between groups	The study found that individuals in the pre‐HD stage have difficulties with egocentric visuospatial working memory but not allocentric working memory. Egocentric memory accuracy was related to the dorsolateral caudate head size, while allocentric memory accuracy was linked to the intermediate and posterior hippocampus volume.
29. Poudel et al. (2015)	22 preHD (10 M; 39.76 ± 9.47) 11 HD (7 M; 51.62 ± 7.26) 20 controls (5 M; 43.53 ± 12.90)	Working memory	N‐BACK task, NART‐2, Edinburgh Handedness Test, UHDRS	Behavioral and functional (fMRI)	Case– control longitudinal study	These results indicate that pre‐HD individuals show increased brain activation in the prefrontal cortex and basal ganglia during working memory tasks, with these changes linked to disease burden and symptom onset timing. Additionally, there is a decrease in functional connectivity between the dorsolateral prefrontal cortex and corpus striatum. These changes may serve as early indicators of functional alterations in pre‐HD individuals.
30. Sarappa et al. (2017)	28 HD (17 M; 41.6 ± 9.6) 11 preHD (5 M; 38.1 ± 7.1) 40 controls (18 M; 37.4 ± 13.5)	Executive functioning	UHDRS fALFF, ReHo	Behavioral and functional (fMRI)	Comparisons between groups	The study highlights that patients with Huntington's disease (HD) exhibit significant alterations in resting‐state brain activity, measured by the fALFF and ReHo of the BOLD signal. Specifically, reductions in neuronal activity were observed in cortical areas involved in executive networks, and altered local synchrony was found in subcortical and cerebellar regions involved in the sensorimotor network.
31. Scahill et al. (2020)	64 pre‐HD‐YAS (30 M,29.0 ± 5.6) 67 HC (28 M,29.1 ± 5.7)	Cognitive flexibility, planning, verbal fluency, emotion recognition, inhibition, attention, learning, memory	CANTAB, EMOTICOM battery, Self‐report questionnaires, Stroop test, SDMT, Verbal fluency test Reinforcement Learning test	Behavioral, functional (MRI), and biofluids analysis	Comparisons between groups	The study indicates that pre‐HD young adults did not exhibit significant differences from the control group in cognitive and neuropsychiatric assessments. Additionally, brain imaging measures showed no significant differences except for a reduced putamen volume in the premanifest phase.
32. Soloveva et al. (2020)	15 pre‐HD (5 M,37.33 ± 10.82) 15 HC (5 M,35.60 ± 10.69)	Visuospatial working memory	MoCA, NART‐2, SDMT, BDI‐ II, UHDRS	Behavioral and functional (fMRI)	Comparisons between groups	Individuals with pre‐HD exhibit deficits in visuospatial working memory, but can compensate by activating different brain areas. Activation of the right insula and left DLPFC seems to facilitate compensation for these deficits. However, contrary to the CRUNCH prediction, pre‐HD individuals did not show increased fMRI activity in the left intraparietal sulcus at lower memory loads. Instead, they displayed decreased activity in this region at higher memory loads, while HC showed increased activity.
33. Unschuld et al. (2013)	41 preHD (15 M; 40.2 ± 10) 12 earlyHD (6 M; 51 ± 8.2) 52 controls (29 M; 39.8 ± 10.2)	Planning and problem solving	ToL	Behavioral and functional (fMRI)	Comparisons between groups	The results show that while performance on the ToL task did not significantly differ between individuals with prodromal HD and HC, changes in the connection of the MPFC were observed across the entire sample in relation to the stage of HD. Further assessment of task‐related BOLD activation could provide a deeper understanding of the neural basis of executive functions in HD. Notably, there was a significant decrease in functional coupling between the MPFC and the left premotor cortex in subjects with HD.
34. Vaca‐Palomares et al. (2017)	23 HD (10 M; 49.6 ± 11.4) 23 controls (10 M; 49.9 ± 10.6)	Flexible executive control and general cognitive functioning	Interleaved pro‐ and antisaccade task, MOCA, UHDRS	Behavioral imaging (MRI)	Comparisons between groups	The study reveals that HD patients have voluntary deficits in controlling saccade inhibition. These deficits are related to atrophy of the white matter. The atrophy of the white matter of the cortico‐basal ganglia in HD interrupts the normal connectivity in a network that controls the inhibitory behavior of the voluntary saccade beyond the indirect path. In vivo measures of white matter atrophy can be a reliable indicator of the progression of cognitive deficits in HD.
35. Vives‐Gilabert et al. (2013)	MEMORY study: 16 pre‐HD (9 M; 35.2 ± 7 8.7) 16 controls (11 M; 36.37 ± 10.4) MOTOR study: 15 pre‐HD (8 M; 36.9 years, range: 26–49) 11 controls (8 M; 36.5 years, range: 23–60) IRRITABILITY study: 16 pre‐HD (8 M; 39.37 ± 7.9) 15 controls (7 M; 40.47 ± 9.4)	Working‐memory and irritability	Sequential finger tapping, working‐memory task, task aiming to induce irritation, UHDRS	Behavioral and functional (fMRI)	Comparisons between groups	This study utilized three fMRI tasks to measure the functional connectivity among different brain regions. Specifically, a verbal working memory task was used to assess the range of cognitive functions affected by HD, including both basic and advanced processes. However, the research found that the functional connectivity graphs generated from fMRI data were inadequate in detecting subtle changes related to the disease, especially when there was significant variability within the group. This limitation persisted regardless of the type of task used.
36. Wolf et al. (2007)	16 preHD: ‐ 8 far preHD (3 M; 33.5 ± 7.9) ‐ 8 close preHD (7 M; 38.1 ± 10.0) 16 controls (11 M; 36.3 ± 11.4)	Alertness, divided attention, verbal and spatial WM, executive function and inhibition processes	UHDRS, BDI, Working memory task, Computerized DA‐test, Computerized WCST, Stroop Word‐Color Interference Test	Behavioral and functional (fMRI)	Comparisons between groups	Results demonstrate that early functional brain changes in pre‐HD subjects may occur in the DLPFC before manifest cortical atrophy, and support a role of this region in the expression of clinical symptoms. Compensatory brain responses in pre‐HD individuals may occur with closer proximity to the onset of manifest clinical symptoms.
37. Wolf et al. (2008a)	16 preHD (9 M; 35.2 ± 8.7) 16 controls (11 M; 36.3 ± 11.4)	Verbal working memory	Edinburgh Handedness Questionnaire, UHDRS, BDI, Cognitive activation task	Behavioral and functional (fMRI)	Comparisons between groups	In individuals with pre‐HD, abnormal functional connectivity in the left DLPFC was detected only under high working memory load conditions. Compared to HC, pre‐HD subjects had weaker connectivity in the left putamen, right anterior cingulate, and left medial prefrontal cortex. Those nearing motor symptom onset also showed reduced connectivity in the right putamen and left superior frontal cortex. The connectivity strength in the left putamen was linked to clinical measures such as CAG repeat length, motor scores, and predicted years to symptom onset.
38. Wolf et al. (2008b)	16 preHD (9 M; 35.2 ± 8.7) 16 controls (11 M; 36.3 ± 11.4)	Verbal working memory	Modified version Sternberg Item Recognition Paradigm, Edinburgh Handedness Questionnaire, UHDRS, BDI	Behavioral and functional (fMRI)	Comparisons between groups	This study discovered that individuals with pre‐HD exhibited reduced context‐dependent contributions in the left putamen, anterior cingulate cortex, and medial prefrontal cortex specifically under conditions of high working memory demands. Additionally, the connectivity strength in the left putamen was associated with various clinical indicators of the disease, such as CAG repeat length and the TMS subscore, as well as the predicted years until the onset of manifest symptoms.
39. Wolf et al. (2009)	12 HD (9 M; 48.4 ± 10.4) 16 controls (11 M; 36.3 ± 11.4)	Alertness, divided attention, verbal and spatial WM, executive function, and inhibition processes	Working memory task, BDI, TFC, UHDRS, Digit and spatial span tasks WCST, Stroop Word‐Color Interference Test, Computerized DA‐test	Behavioral and functional (fMRI)	Comparisons between groups	According to the study, during verbal working memory manipulation, certain cortical and subcortical areas show less activation in people who demonstrate a workload‐dependent deficit in verbal working memory and delayed recall. Particularly HD individuals show less activation in the right cerebellum, left inferior parietal cortex, left putamen, and left dorsolateral and ventrolateral prefrontal cortex‐but only when they are under a lot of working memory strain.
40. Wolf et al. (2011)	13 preHD (6 M; 34.8 ± 8.9) 13 controls (9 M; 39.2 ± 8.9)	Alertness, divided attention, verbal and spatial WM, executive function and inhibition processes	UHDRS, HAMD, BDI, Computerized DA‐test, Computerized version of the WCST, Stroop Word‐Color Interference Test, Modified version of the Sternberg Item Recognition Paradigm	Behavioral and functional (fMRI)	Longitudinal study Comparisons between groups	The study discovered that the left DLPFC, essential for working memory processing, consistently showed different activity levels in HD expansion mutation carriers, even though their cognitive performance remained normal. Additionally, it found that pre‐HD subjects exhibited lower brain activation in specific frontostriatal regions during selective attention tasks compared to HC. Furthermore, decreased striatal activation correlated with longer reaction times and closeness to the onset of the disease.
41. Wolf et al. (2012a)	18 preHD (8 M; 36.3 ± 9.0): ‐ 9 preHDfar (2 M; 32.9 ± 7.0) ‐ 9 preHDnear (6 M; 39.8 ± 9.8) 18 controls (9 M; 37.2 ± 10.3)	Attentional processing, alertness	UHDRS, BDI, HAMD, Semistructured psychiatric interview	Behavioral and functional (fMRI)	Comparisons between groups	The results of this study reveal that decreased striatal activation is linked to slower reaction times and closer proximity to disease onset. Pre‐HD patients nearing disease onset also exhibit reduced functional connectivity in motor regions compared to both HC patients and pre‐HD patients who are farther from onset.
42. Wolf et al. (2012b)	18 preHD (8 M; 36.3 ± 9.0) 18 controls (9 M; 37.2 ± 10.3)	Attentional function	Attention task, UHDRS, BDI, HAMD	Behavioral and functional (fMRI)	Comparisons between groups	The study shows highlights changes in the Default Mode Network (DMN) functionality in individuals with pre‐HD as observed through fMRI during an attention task. Pre‐HD subjects exhibit reduced functional connectivity within the DMN, specifically in the PCC, the amPFC, and the left IPL.
43. Wolf et al. (2014)	13 preHD (6 M; 34.8 ± 8.9) 13 controls (9 M; 39.2 ± 8.9)	Attention, Working‐memory, task‐related deactivation dynamics	UHDRS, BDI, HAMD	Behavioral and functional (fMRI)	Longitudinal study Comparisons between groups	Functional connectivity analyses within two distinct task‐negative networks revealed differences in the PCC and left anterior prefrontal cortex, both at baseline and follow‐up, without evidence of progression over time. Additionally, a relationship emerged between the connectivity of the dorsal cingulate cortex and motor function, suggesting that “task‐negative” activity could provide further insights into time‐sensitive neural and functional processes in preHD.
44. You et al. (2014)	15 pre‐HD (45.6 ± 12.0) 42 HC (47 ± 14.2)	Executive functions working memory, cognitive control, fluency	UHDRS, MoCA, CAPs, NIH EXAMINER	Behavioral and functional (3T MR scanner)	Comparisons between groups	The pre‐HD scored significantly lower on the working memory score. The executive composite positively correlated with striatal volumes, and the working memory score negatively correlated with disease burden. The cognitive control and fluency scores did not differ between the groups or correlate significantly with the disease markers.

Abbreviations: amPFC, middle anterior prefrontal cortex; AVLT, auditory verbal and learning test; BDI, Beck's Depression Inventory; BOLD, blood oxygenation level dependent; CANTAB, Cambridge Neuropsychological Test Automated Battery; CAPs, CAG‐Age Product Scaled; CRUNCH, Compensation‐Related Utilization of Neural Circuits Hypothesis; DBS, deep brain stimulation; DLPFC, dorsolateral prefrontal cortex; DWI, Diffusion‐weighted imaging; EEG, electroencephalogram; ERPs, event‐related potentials; FAB, Frontal Assessment Battery; fALFF, Fractional Amplitude of Low Frequency Fluctuations; fMRI, functional magnetic resonance imaging; FrSBe, Frontal System Behavior Scale; HAMD, Hamilton Depression Rating Scale; HC, healthy controls; HD, Huntington's disease; HVLT, Hopkins Verbal Learning Test‐Revised; IED, intra‐extra dimensional set‐shift; IPL, inferior parietal lobe; IS, Independence Scale; LRP, Lateralized Readiness Potential; MMNm, Magnetic Mismatch Negativity; MMSE, Mini‐Mental State Examination; MoCA, Montreal Cognitive Assessment; MPFC, medial prefrontal cortex; MRI, Magnetic Resonance Imaging; NART, National Adult Reading Test; PCC, posterior cingulate cortex; PFC, prefrontal cortex; pre‐HD, presymptomatic HD; qEEG, Quantitative Electroencephalography; ReHo, Regional Homogeneity; SART, Sustained Attention to Response Task; SDMT, Symbol Digit Modalities Test; TFC, Total Functioning Capacity; TISL, training‐independent sensory learning; TMSs, transcranial magnetic stimulation; TMT, trail making test; ToL, Tower of London; TOPF‐UK, test of premorbid functioning; UHDRS, unified Huntington's disease rating scale; VLMT, Verbal learning Memory Test; WAIS, Wechsler Adult Intelligence Scale; WAIS, Wechsler Adult Intelligence Scale; WCST, Wisconsin Card Sorting Test; WMS‐R, Wechsler Memory Scale‐Revised; YMRS, Young Mania Rating Scale.

We have provided below a breakdown of the studies by executive subdomain, and within each section, we have described the studies according to disease stage (from presymptomatic to symptomatic). In particular, the following executive subdomains were taken into account:

‐ Decision making: the cognitive process of selecting a course of action among multiple alternatives through the evaluation of available information, potential consequences, and expected outcomes;
‐ Working memory: the cognitive system responsible for the temporary storage and manipulation of information necessary for complex cognitive tasks such as reasoning, learning, and comprehension;
‐ Cognitive flexibility, set/task switching, and dual‐task abilities: executive functions involved in adapting to changing demands, shifting between tasks or mental sets, and managing multiple tasks simultaneously through efficient cognitive control;
‐ Inhibitory control/interference: the executive function that enables the suppression of automatic or irrelevant responses and resistance to interference in order to maintain goal‐directed behavior;
‐ Processing speed/attention: the cognitive ability to efficiently process information and maintain focused, sustained attention to support rapid and accurate task performance;
‐ Planning/problem solving: the executive function that involves generating, organizing, and implementing strategies to achieve goals and resolve novel or complex situations.

### 3.2 Decision Making

Across studies, decision‐making abilities remain relatively preserved during the pre‐HD stage but deteriorate following motor onset. Some studies report that pre‐HD individuals show normal or cautious decision patterns, slower responses, and a tendency to avoid risky choices, suggesting compensatory control strategies (Adjeroud et al. 2017; D'Aurizio et al. 2019). In contrast, manifest and early HD patients show deficits in decision making under ambiguity, increased impulsivity, and greater temporal discounting, reflecting a stronger preference for immediate rewards and impaired feedback monitoring (Adjeroud et al. 2017; El Haj et al. 2023). Other studies confirm altered inhibitory control and a bias toward high‐risk or disadvantageous options despite explicit awareness of negative outcomes, suggesting impaired reward valuation (Galvez et al. 2017; Holl et al. 2013; Van Wouwe et al. 2016). Collectively, these findings support a stage‐dependent decline in decision‐making efficiency, with progressive disruption of cognitive control and reward‐based learning mechanisms in HD.

### 3.3 Working Memory

Evidence from behavioral and neuroimaging studies indicates that WM alteration appear in the pre‐HD stage and progressively worsen after motor onset. In prodromal HD, impairments in visuospatial WM are already detectable compared to healthy controls (HC; Dumas et al. 2012). In the early manifest stage of HD, additional deficits emerge in WM storage capacity and spatial object WM, with a progressive longitudinal decline as the disease advances (Bachoud‐Lévi et al. 2001; Finke et al. 2006, 2007; Watkins et al. 2000).

Regarding neuroimaging studies, several evidence shows that frontostriatal and prefrontal dysfunctions begin in pre‐HD stage and progressively intensify after motor onset. In pre‐HD individuals, functional MRI studies consistently reveal reduced activation of the left dorsolateral prefrontal cortex (DLPFC) and altered connectivity within the frontostriatal circuit during WM tasks, even when behavioral performance remains normal (Wolf et al. 2008a, 2007, 2011, 2008b). Decreased connectivity involving the putamen, anterior cingulate, and medial prefrontal cortex correlates with genetic burden and proximity to clinical onset (Wolf et al. 2008a). Longitudinal fMRI findings show that increased activation in the left DLPFC and medial frontal cortex, along with further recruitment of the bilateral caudate and putamen in pre‐HD, reflects early neural adaptation preceding overt decline (Poudel et al. 2015). In pre‐HD individuals, fMRI graph‐based analyses during WM tasks revealed no significant differences from HC. Standard network metrics failed to detect subtle preclinical alterations, indicating limited sensitivity for detecting changes at the presymptomatic stage (Vives‐Gilabert et al. 2013).

Further studies demonstrate that WM capacity is associated with specific connectivity patterns between prefrontal, caudate, and occipital regions (Harrington et al. 2014). Despite preserved accuracy, subtle visuospatial and egocentric/allocentric WM impairments emerge early, paralleling structural changes in the caudate nucleus and hippocampus (Possin et al. 2017; Soloveva et al. 2020; You et al. 2014). These findings suggest that early cognitive alterations in pre‐HD are linked to striatal atrophy and compensatory DLPFC–insula recruitment, supporting transient neural reorganization.

In contrast, manifest HD patients exhibit clear deficits in WM accompanied by reduced activation of the DLPFC, ventrolateral PFC, inferior parietal cortex, putamen, and cerebellum under high cognitive load (Aron et al. 2003; Wolf et al. 2009). Structural MRI consistently shows caudate and thalamic atrophy (Kim et al. 2017), indicating basal‐ganglia‐thalamo‐cortical disconnection as a core substrate of WM dysfunction (Kassubek et al. 2005; Martinez‐Horta et al. 2020; Peinemann et al. 2005; Vaca‐Palomares et al. 2017).

Across disease stages, white matter and myelin abnormalities emerge as critical contributors to cognitive decline. Reduced fiber integrity and myelin fraction within dorsolateral and orbitofrontal regions correlate with poorer WM performance, even before motor onset (Casella et al. 2021; Matsui et al. 2014, 2015). Together, these findings depict a continuum from functional reorganization in prodromal HD to structural disconnection and network failure in manifest HD, mirroring the progressive breakdown of frontostriatal, thalamic, and white matter pathways underlying WM dysfunction.

### 3.4 Cognitive Flexibility, Set/Task Switching, Dual Task

Across the disease continuum, converging evidence shows a progressive decline in cognitive flexibility, set/task shifting and dual task from the premanifest to the manifest stages. In pre‐HD, impairments are subtle and domain‐specific, mainly involving cognitive flexibility and task switching (Heim et al. 2020; Larsson et al. 2008; Migliore et al. 2019; O'Rourke et al. 2011). These alterations intensify as individuals approach clinical onset, with near‐to‐onset carriers showing slower task switching, and decreased fluency relative to HC (Unmack Larsen et al. 2015). Neuroimaging findings confirm this, showing altered connectivity in the ventrolateral prefrontal cortex (VLPFC), which suggests compensatory circuit reorganization during the presymptomatic stage (Langley et al. 2021). Moreover, studies in the same disease stage have highlighted a reduction in putamen volume (Scahill et al. 2020).

In the manifest phase, deficits become more widespread and patients in early HD stage show marked slowing of attentional shifts and impaired disengagement from previous stimuli (Couette et al. 2008; A. Lawrence 1998; A. D. Lawrence et al. 1996; Sprengelmeyer et al. 1995). Performance in dual‐task paradigms further demonstrates impaired automaticity and limited attentional resources, resulting in greater interference between cognitive and motor processes (Delval et al. 2008; Purcell et al. 2020; Thompson et al. 2010; Vaportzis et al. 2015b, Vaportzis et al. 2015a). These findings highlight that executive–motor coupling is disproportionately affected in HD, particularly during gait and coordination tasks requiring simultaneous cognitive effort.

Structural and functional imaging supports these behavioral findings. Thalamic and basal ganglia atrophy correlate with loss of cognitive flexibility (Aron et al. 2003; Casella et al. 2021; Kassubek et al. 2005; Martinez‐Horta et al. 2020; Peinemann et al. 2005; Vaca‐Palomares et al. 2017), while white matter degeneration in dorsolateral and orbitofrontal regions further contributes to disrupted frontostriatal communication, already detectable in the prodromal stage (Matsui et al. 2014, 2015). Neurophysiological investigations using transcranial magnetic stimulation revealed no significant differences in cortical excitability measures between HD patients and HC and no clear association with cognitive flexibility, underscoring the complexity of cortical dysfunction and the need for multimodal approaches to clarify its link with executive decline (Kamble et al. 2018). Deficits in motor–cognitive integration are linked to altered connectivity in the right inferior parietal gyrus, right thalamus, left anterior cingulate cortex, and sensorimotor areas reinforcing the role of large‐scale network disconnection in HD‐related executive dysfunction (Harrington et al. 2015; Koenig et al. 2014).

### 3.5 Inhibitory Control/Interference

Converging evidence indicates that inhibitory control in HD follows a progressive trajectory from the premanifest to the manifest stage. In pre‐HD individuals, computational and behavioral approaches reveal early alterations in inhibitory processes, even before motor onset. Modeling studies suggest that parameters related to executive control predict HD in its presymptomatic phase, while selective deficits in response inhibition emerge only after symptom onset (Wiecki et al. 2016). Behavioral paradigms confirm specific impairment in inhibiting automatic visuospatial responses and subtle impairments in attentional control and orienting (Farrow et al. 2006, Farrow et al. 2007). Large‐scale studies, such as PREDICT‐HD, demonstrate that cognitive decline—for example, in inhibitory control—can be reliably detected years before diagnosis, progresses independently of motor signs, and becomes more pronounced near clinical onset (Stout et al. 2011).

In manifest and early manifest HD, deficits extend across multiple cognitive domains, with response inhibition showing the most pronounced longitudinal decline (J. Snowden et al. 2001; Sprengelmeyer et al. 1995). Specific impairments in visuospatial attention and oculomotor inhibition have also been identified, reflecting underlying inhibitory dysfunction (Fielding et al. 2006).

Electrophysiological markers such as the N1/LRP (reflecting early perceptual and motor preparation processes; Beste et al. 2008a; Beste, Saft, Güntürkün, et al. 2008b), P300 (indexing attentional allocation and stimulus evaluation; Hart et al. 2012; Jurgens et al. 2011), and delta‐Ne (associated with error monitoring and cognitive control; Beste et al. 2007) show progressive alterations in pre‐HD correlated with basal ganglia atrophy. Additional ERP studies highlight attenuated flexible response adaptation and disrupted connectivity between prefrontal and striatal regions (Beste et al. 2011, 2012, 2015), supporting a functional disconnection model of early executive decline.

Structural and functional imaging studies further corroborate these findings. Altered connectivity within the right inferior parietal gyrus, thalamus, anterior cingulate cortex, and sensorimotor areas correlates with inhibitory dysfunction (Harrington et al. 2015; Koenig et al. 2014). Early reductions in activity within the precuneus and superior frontal cortex have been associated with difficulty in inhibitory processes (Sarappa et al. 2017). In addition, white matter disconnection within the inferior fronto‐occipital fasciculus and corona radiata impairs the voluntary inhibition of saccades, reinforcing the role of structural network deterioration as a driver of executive deficits (Casella et al. 2021; Vaca‐Palomares et al. 2017).

### 3.6 Processing Speed/Attention

In pre‐HD individuals, impairments in psychomotor speed and attention emerge years before diagnosis (Hart et al. 2013, 2014; J. S. Snowden et al. 2002; Verny et al. 2007), worsening in those nearer to onset. Oculomotor dysfunctions correlate with reduced performance on psychomotor speed tasks, underscoring the link between motor and cognitive control systems (Carvalho et al. 2016). Dual‐task interference and reduced processing speed further indicate early prefrontal dysfunction related to disease burden (Paz‐Rodríguez et al. 2024; Reyes et al. 2021).

In manifest HD patients attention impairments and slower information processing speed become pervasive, with a progressive deterioration over time (Bachoud‐Lévi et al. 2001; Ho et al. 2003; Julayanont et al. 2020; A. D. Lawrence et al. 2000; Lemiere et al. 2004; Sprengelmeyer et al. 1995). Conversely, Maurage et al. (2017) showed that manifest HD does not lead to a global attentional deficit but rather to a specific impairment for the executive control of attention. Eye‐movement studies reveal prolonged saccadic latencies and altered orienting, implicating greater difficulty in directing visual attention (Georgiou‐Karistianis et al. 2012; Tsai et al. 1995).

Neurophysiological and neuroimaging findings converge on frontostriatal and prefrontal disconnection as the substrate of executive decline. EEG/qEEG and MEG studies show altered cortical power spectra and impaired involuntary attention in both pre‐HD and manifest HD, reflecting dysfunction of cortico‐striatal networks and dopaminergic modulation (Cheng et al. 2014; Odish et al. 2018). Altered dopamine cortical metabolism further contributes to cognitive dysfunction: PET studies revealed reduced D_2_ receptor availability in striatal and cortical regions of HD patients, which correlates with poorer attentional and executive performance across disease stages (Pavese et al. 2010). fMRI studies reveal reduced activation and connectivity within the frontostriatal and default mode networks during attentional tasks—particularly in the anterior middle prefrontal cortex, anterior and posterior cingulate, inferior parietal lobule, striatum and sensorimotor regions—with abnormalities that increase as individuals approach disease onset and show a progressive longitudinal decline (Domínguez D et al. 2017; Ferraro et al. 2014; Koenig et al. 2014; Wolf et al. 2012a, 2011, 2012b, 2014). Finally, structural MRI studies have reported early frontal white matter degeneration and reduced fiber integrity in the dorsolateral and orbitofrontal regions, which correlate with slower information processing speed and attentional deficits, supporting white matter disruption as an early biomarker (Matsui et al. 2014, 2015).

### 3.7 Planning/Problem Solving

In pre‐HD individuals, subtle impairments in planning, strategy use, and problem solving are already evident and correlate with disease proximity and structural changes in frontostriatal circuits (Allain et al. 2005; Horta‐Barba et al. 2021; Papp et al. 2013; Unschuld et al. 2013; Watkins et al. 2000). Tasks such as the Tower of London and arithmetic problem solving reveal early frontal–executive deficits, even in the absence of overt motor signs (Allain et al. 2005; Horta‐Barba et al. 2021; Mörkl et al. 2016). Conversely, the Wisconsin Card Sorting Test—a task used to evaluate planning and abstract reasoning—often fails to detect differences during the premanifest phase, except in individuals who later convert to the manifest stage, highlighting its limited sensitivity to early decline (Brandt et al. 2008; Campodonico et al. 1996). Studies on counterfactual reasoning confirm that higher order executive functions deteriorate as the disease advances (Solca et al. 2015).

### 3.8 Executive Dysfunctions and Correlation With Other Domains

Executive impairments in prodromal HD emerge more than a decade before motor onset, with reduced performance on general intelligence and executive measures (Robins Wahlin et al. 2010). Screening tools such as the Frontal Assessment Battery show utility for distinguishing HD patients from HC and for monitoring cognitive decline over time (Rodrigues et al. 2009; Solca et al. 2022). Deficits in self‐awareness (anosognosia) are frequent in manifest HD and correlate with poorer executive functioning and with greater caregiver burden (Hoth et al. 2007; Wibawa et al. 2020). Motor and executive dysfunctions appear closely interrelated. Hypokinesia and impaired voluntary movement are associated with reduced executive functioning, independent of chorea or apathy, highlighting a shared neurofunctional substrate (Klempíř et al. 2009; Reedeker et al. 2010). Moreover, executive impairments correlate with gait and balance disturbances, underscoring the executive contribution to motor disability in HD (Kloos et al. 2017). Finally, obsessive–compulsive symptoms are reported in over half of manifest HD patients and are linked to poorer executive performance, supporting the role of basal ganglia dysfunction in both behavioral and cognitive manifestations of the disease (Anderson et al. 2001).

### 3.9 Functional Impact

Executive dysfunction significantly impacts everyday functioning in HD, affecting autonomy in tasks such as driving, financial management, and employment. Both cognitive and motor deficits independently predict loss of functional abilities, with psychomotor slowing and attentional deficits as key contributors (Eddy and Rickards 2015; Rothlind et al. 1993; Van Liew et al. 2013). In manifest HD, reduced executive control is strongly associated with unemployment, apathy, and impulsivity (Atkins et al. 2024; Jacobs, Hart, and Roos 2018b; Johnson et al. 2017; McLauchlan et al. 2019).

Regarding driving performance, both pre‐HD and manifest HD individuals show compromised control, slower speed regulation, and poorer reaction times compared to HC, primarily linked to executive rather than motor dysfunction (Devos et al. 2012, 2014; Hennig et al. 2014; Jacobs et al. 2018a). Slower processing speed, together with inhibitory dyscontrol and divided attention impairments, is the strongest predictor of unsafe driving, supporting the inclusion of detailed neuropsychological screening in clinical evaluations of HD drivers (Beglinger et al. 2012).

**FIGURE 2:**
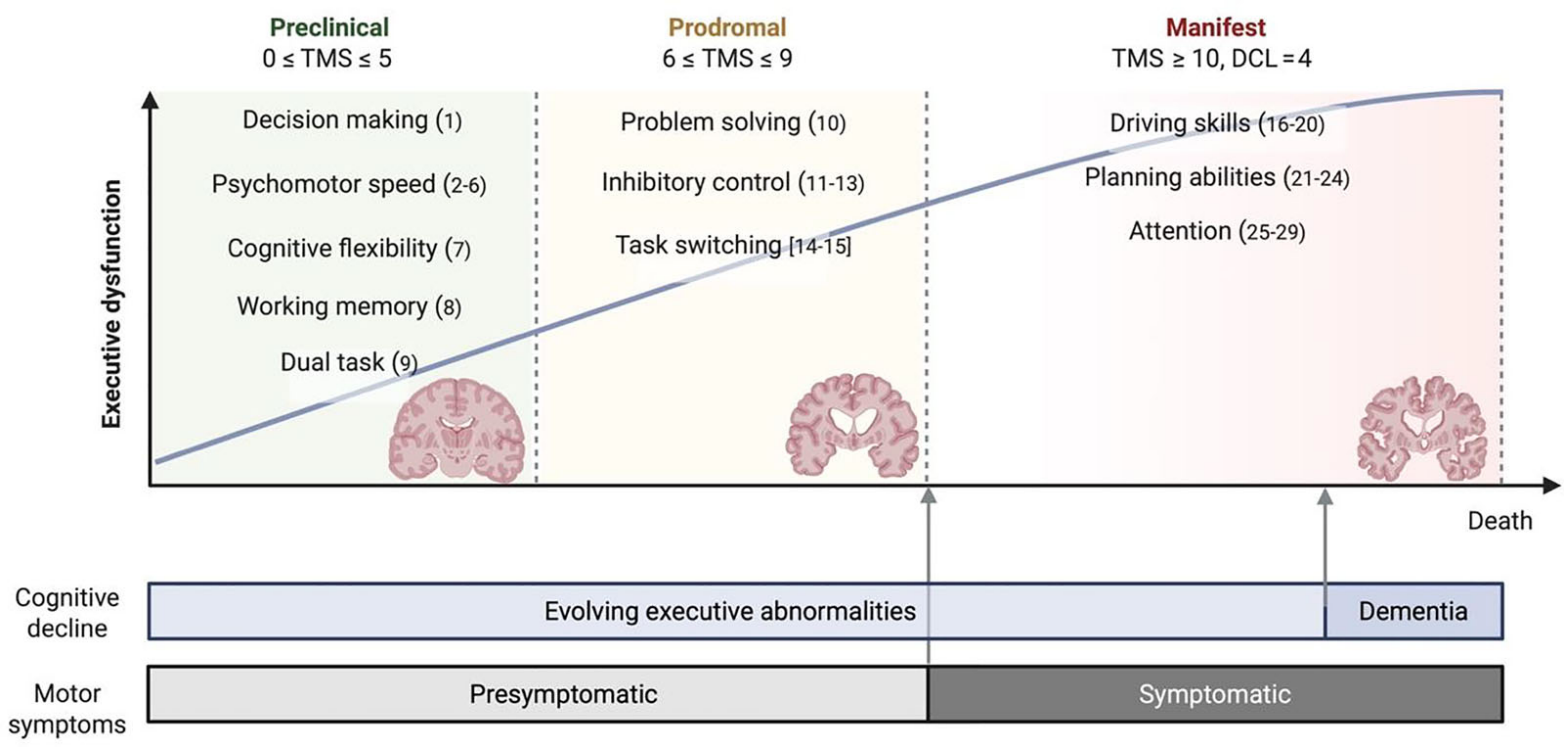
The figure illustrates the presence of specific executive deficits according to the disease stage. The blue line represents the progressively worsening trajectory of executive dysfunction overtime. The period before the motor diagnosis of Huntington's disease is termed “premanifest.” During the “preclinical” period, no motor signs are present. In “prodromal” Huntington's disease, subtle motor signs appear. Manifest Huntington's disease is characterized by significant motor symptoms. Only the bibliographic references of studies that have identified the specific executive deficit in the earliest stage of the disease are reported. Other studies have also observed the same deficits at different disease stages; however, they have not been included in the figure to prevent excessive information overlap. Each specific number listed in the figures corresponds to the full bibliographic reference listed in the Supporting Information. The figure was created with BioRender.com.

**FIGURE 3:**
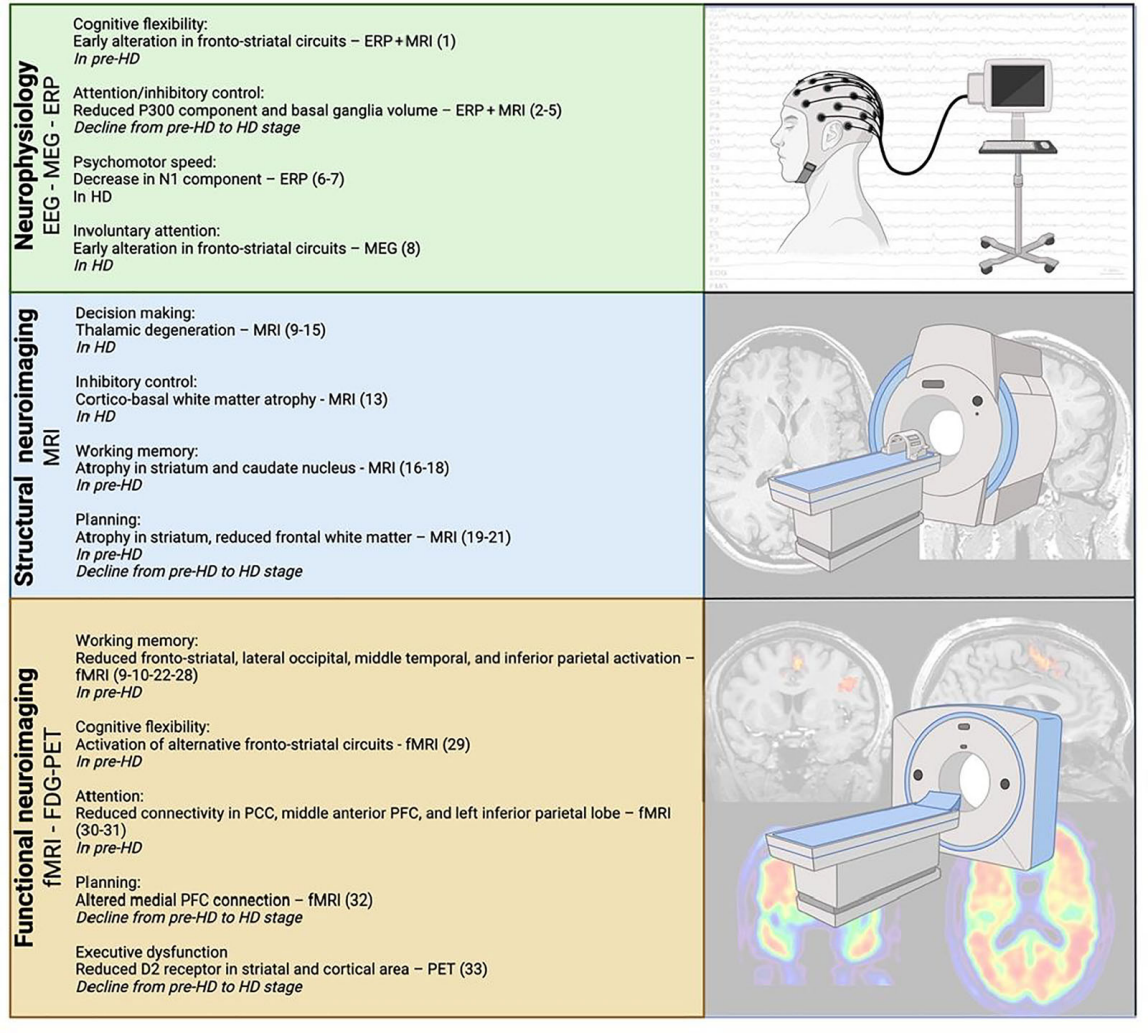
Executive dysfunction and brain correlates according to the different investigative methodologies considered. Each specific number listed in the figures corresponds to the full bibliographic reference listed in the Supporting Information. The figure was created with BioRender.com.

Main evidence of executive dysfunction in pre‐HD and manifest HD are summarized in Figure 2. Moreover, main evidence of executive dysfunction and brain correlate are summarized in Figure 3.

## 4 Discussion

The present review is updated to November 2024, provides a systematic mapping between executive function domains and their underlying neural circuits, and integrates multimodal findings from neuroimaging and neurophysiological research with clinical and functional outcomes. Moreover, by emphasizing longitudinal data, it delineates how executive dysfunction emerges and progresses across the clinical spectrum of HD, highlighting its potential as an early marker and therapeutic target. Compared with previous syntheses (Cavallo et al. 2022; Papoutsi et al. 2014), this review thus provides a more comprehensive and integrative perspective on the neural and functional correlates of executive impairment in HD.

More specifically, our review showed that executive deficits are present even in the early stages, since the presymptomatic stage of the disease before the onset of motor symptoms, and progressively worsen from the prodromal to the symptomatic stage (see Figure 2 for more details).

We observed that in the pre‐HD stage a number of studies consider their findings as evidence of the presence of subtle cognitive changes many years before the onset of motor symptoms, and some suggest that executive functions are good predictors of cognitive impairment and prognostic indicators of the future clinical course of the disease. Executive functions impairment can be detected 9–15 years before the estimated onset of clinical diagnosis and become more pronounced as the disease progresses (O'Rourke et al. 2011; see Figure 2 for more details). Moreover, several findings demonstrated that executive functions were correlated with mild “soft” motor symptoms, particularly in individuals nearing the clinical onset of HD. This suggests a gradual, progressive evolution of the executive dysfunction (Hart et al. 2013, 2014; J. S. Snowden et al. 2002). Several executive domains were impaired in HD, including information processing speed (Carvalho et al. 2016; Hart et al. 2014; Paz‐Rodríguez et al. 2021; J. S. Snowden et al. 2002; Verny et al. 2007), decision making (D'Aurizio et al. 2019), WM (Dumas et al. 2012), verbal fluency (Larsson et al. 2008), and task switching (Migliore et al. 2019; O'Rourke et al. 2011). Parameters related to executive control have been shown to predict HD before the onset of motor symptoms (El Haj et al. 2023; Farrow et al. 2006, 2007), whereas impairments in attention (Bachoud‐Lévi et al. 2001; Couette et al. 2008; Georgiou‐Karistianis et al. 2012; Ho et al. 2003; Lemiere et al. 2004) and planning abilities (Allain et al. 2005; Brandt et al. 2008; Solca et al. 2015; Watkins et al. 2000) mainly emerged only after motor symptoms were observed. Also, oculomotor dysfunctions were correlated with cognitive functioning, particularly in tasks requiring psychomotor speed and ability to handle multiple tasks simultaneously (Carvalho et al. 2016; Farrow et al. 2006, 2007). Numerous studies showed that early decision‐making difficulties often had an impact on daily living skills, such as driving or making financial decisions (Beglinger et al. 2012; D'Aurizio et al. 2019; Devos et al. 2012, 2014; Hennig et al. 2014; Horta‐Barba et al. 2021; Jacobs et al. 2018a). Thus, executive dysfunction in HD is not merely a laboratory finding, but a determinant of everyday quality of life—affecting the ability to drive, work, and engage appropriately in financial affairs. Recognizing and addressing early these issues is crucial for patient care, as interventions that support executive function may prolong independence in daily living.

In manifest HD, both motor and cognitive impairments become increasingly pronounced, with executive dysfunction emerging as a key determinant of patients’ functional decline and loss of autonomy (Eddy and Rickards 2015; Jacobs, Hart, and Roos 2018b; Rothlind et al. 1993; Van Liew et al. 2013). As cognitive resources become more limited, patients show greater difficulty in managing competing tasks, suggesting reduced capacity for divided attention and cognitive control (Delval et al. 2008; Sprengelmeyer et al. 1995; Thompson et al. 2010).

These cognitive and motor impairments have important consequences for daily functioning (Beglinger et al. 2012; Devos et al. 2012, 2014; Hennig et al. 2014; Jacobs, Hart, and Roos 2018b). The interplay between motor and cognitive domains is further supported by findings linking hypokinesia and balance deficits to executive dysfunction, independently of chorea or apathy (Klempíř et al. 2009; Kloos et al. 2017; Reedeker et al. 2010). Overall, the manifest phase represents a multidimensional deterioration in which executive dysfunction amplifies the impact of motor and behavioral symptoms, accelerating the decline in autonomy and quality of life. Its progressive spread to other cognitive domains—such as memory and language—suggests a network‐level breakdown that integrates cognitive and motor control failure within shared frontostriatal and cortical systems. However, the available evidence indicates that executive dysfunction, motor impairments and behavioral alterations co‐occur and correlate. Their association is most plausibly explained by shared degeneration of frontostriatal, thalamocortical, and sensorimotor networks that jointly support both cognitive/behavioral control and motor coordination. Thus, difficulties in executive functioning, mobility and behavioral control should be viewed as parallel manifestations of common underlying neuropathology rather than one directly driving the other.

The synthesis of neurophysiological and neuroimaging evidence provides a coherent framework for understanding the neural mechanisms underlying executive dysfunction in HD. Across modalities, findings converge on the early disruption of frontostriatal and frontocortical networks, emphasizing that alterations in these circuits precede the onset of overt motor symptoms (see Figure 3 for more details). ERP and qEEG studies suggest that early deficits in cognitive control reflect compromised neural efficiency in frontostriatal pathways and may represent early electrophysiological markers of disease progression (Beste et al. 2008a, 2007; Beste, Saft, Güntürkün, et al. 2008b; Hart et al. 2012; Odish et al. 2018).

fMRI evidence indicates reduced activation of key executive regions (DLPFC, ventrolateral PFC, putamen, and anterior cingulate cortex), with connectivity disruptions in both frontostriatal and default mode networks correlating with genetic burden and proximity to clinical onset (Langley et al. 2021; Poudel et al. 2015; Wolf et al. 2011, 2007; see Figure 3 for more details). These findings highlight the functional reorganization that may initially compensate for neuronal loss but ultimately fails as disease advances.

Structural MRI studies reinforce this interpretation by linking early atrophy in the caudate, putamen, thalamus, and associated white matter tracts to declining executive control, suggesting a progressive collapse of the structural scaffolding supporting cognitive flexibility (Casella et al. 2021; Unschuld et al. 2013). Similarly, PET evidence points to dopaminergic dysfunction within striatal and cortical circuits, aligning with attentional and executive impairments and implicating neurotransmitter imbalance in the early cognitive phenotype of HD (Pavese et al. 2010). MEG and TMS findings further indicate disrupted cortico‐striatal communication and altered cortical excitability, though their clinical correlates require clarification (Cheng et al. 2014; Kamble et al. 2018).

The present synthesis suggests that executive dysfunction in HD cannot be interpreted as a unitary deficit but rather reflects the progressive breakdown of specific large‐scale control networks that implement classical executive processes described in cognitive psychology. In particular, the observed early impairments in inhibition, WM updating, and cognitive flexibility map closely onto the core executive components proposed in the influential unity‐and‐diversity model of executive functions (Friedman and Miyake 2017). From a theoretical perspective, these deficits can also be interpreted within the Supervisory Attentional System (SAS) framework (Norman and Shallice 1986), which posits that goal‐directed behavior depends on a control system that biases lower level action schemas in situations requiring planning, inhibition, or conflict resolution. The early alterations in response inhibition, task switching, and monitoring observed in pre‐HD are consistent with a disruption of this supervisory control system due to frontostriatal disconnection. Within WM theory, early deficits in updating and manipulation are consistent with dysfunction of the central executive component of Baddeley's multicomponent model (Baddeley 2003, 2012), which relies heavily on dorsolateral prefrontal and striatal interactions. Neuroimaging evidence reviewed here indicates that these circuits are among the earliest affected in HD, providing a direct neural substrate for the observed executive impairments. Importantly, functional neuroimaging studies in premanifest HD frequently show increased recruitment of prefrontal cortical regions during executive tasks, even when behavioral performance is still preserved. This pattern is consistent with network compensation models, in which frontal cortical hyperactivation temporarily offsets striatal and white matter degeneration (Gregory et al. 2018; Reuter‐Lorenz and Cappell 2008). As the disease progresses, however, this compensatory capacity collapses as structural and functional disconnection within frontostriatal, thalamocortical, and frontoparietal circuits becomes more pronounced, leading to genuine network failure and overt executive dysfunction.

By integrating behavioral, neuroimaging, and neurophysiological evidence across disease stages, this review moves beyond domain‐specific descriptions of executive impairment and provides a stage‐sensitive, circuit‐based model of executive dysfunction in HD. Our synthesis demonstrates how early executive alterations map onto specific patterns of frontostriatal disconnection and how these relationships evolve longitudinally (see Figure 2). Importantly, our results show that different executive components (e.g., inhibition, WM, cognitive flexibility) have distinct temporal trajectories and neural correlates, rather than declining uniformly. This refines the concept of “executive dysfunction” in HD and supports a fractionated, network‐based view of cognitive decline.

The present findings have direct clinical implications. First, the consistent sensitivity of executive measures to premanifest and prodromal HD suggests that detailed executive testing should be incorporated into routine monitoring of gene‐expansion carriers, even in the absence of motor symptoms. Second, the strong correspondence between executive performance and frontostriatal imaging and electrophysiological markers supports their use as composite biomarkers for disease staging and progression. Such multimodal markers may be particularly valuable in clinical trials aimed at delaying disease onset or slowing cognitive decline, where traditional motor outcomes may lack sensitivity in early stages. Moreover, identifying which executive components decline earliest may guide the development of targeted cognitive rehabilitation and pharmacological interventions.

Future studies should prioritize longitudinal, multimodal designs combining cognitive testing, neuroimaging, and electrophysiology to model individual trajectories of executive decline. The application of computational and network‐based approaches may further clarify how specific circuit disruptions give rise to different executive profiles. Finally, greater standardization of executive function measures across studies would improve comparability and facilitate meta‐analytic approaches. Finally, integrating executive markers with genetic indicators may allow the development of precision‐medicine strategies for cognitive outcomes in HD.

### 4.1 Limitations

Several limitations need to be acknowledged in this review due to the inherent heterogeneity among the studies included. First, the small sample sizes across various studies limit the generalizability and statistical power of the findings. Regarding the duration of follow‐up studies, which ranged from 1 to 10 years, we did not focus on the specific duration. As with sample size, short follow‐up periods may limit the ability to effectively investigate cognitive changes in HD. Additionally, there is substantial variability and heterogeneity among studies in distinguishing between the presymptomatic and manifest phases of the disease. Typically, two parameters are considered: TMS and DCL, but the reference cut‐off values often differ across studies. This variability made comparative studies unreliable. Moreover, the utilized cognitive tasks varied widely among studies, making it challenging to directly compare cognitive outcomes.

Given the significant heterogeneity among the included studies in terms of design, participant characteristics (e.g., premanifest vs. symptomatic HD cohorts), outcome measures (various executive function tasks), and statistical reporting approaches, we did not perform a formal meta‐analysis of the results. Such diversity across studies would have precluded any meaningful quantitative synthesis; accordingly, our review provides a qualitative narrative summary rather than pooled effect size estimates. We acknowledge that the absence of calculated effect sizes (e.g., Cohen's *d*, Hedges’ *g*) and formal publication bias analyses (e.g., funnel plots, Egger's test) is a limitation of this review. Consequently, while this review offers valuable insights into executive dysfunctions across different stages of HD, as well as their correlation with structural and functional brain changes, caution is warranted when interpreting and generalizing the findings due to these inherent limitations and variations. Future systematic reviews aiming to conduct a meta‐analysis on this topic may benefit from the involvement of additional statistical expertise and thorough methodological peer review to ensure robust quantitative analysis. Moreover, although this review was based on studies retrieved from two major databases (PubMed and PsycINFO), which still represents a limitation in terms of comprehensive coverage, the use of complementary biomedical and psychological databases helped to reduce the risk of selection bias. Future reviews should adopt broader multidatabase search strategies to ensure maximal completeness. Again, a further limitation concerns the absence of a formal risk‐of‐bias tool (e.g., NOS, ROBINS‐I, RoB2). Although a structured qualitative framework was employed to minimize potential bias through predefined methodological criteria and dual independent evaluation, this approach lacks the standardized quantification provided by formal instruments and may therefore reduce comparability with other systematic reviews. Finally, this review deliberately excluded cross‐disease comparative studies to preserve specificity regarding executive dysfunction in HD. However, this approach limits the broader interpretative context, as cross‐disorder comparisons (e.g., with PD or FTD) could further clarify shared versus HD‐specific mechanisms of executive decline.

## 5 Conclusions

In conclusion, the studies analyzed in this review highlight a broad spectrum of impairments in executive functions. These difficulties emerge in the early stages of the disease, with subtle changes occurring many years before the onset of motor symptoms, and persist, worsening as the disease progresses to the manifest phase. These deficits are associated with both structural and functional brain changes that underscore the progressive nature of the damage as the disease advances. In the presymptomatic phase of the disease, the predominantly affected brain structures and networks are those within the fronto‐subcortical circuits. Executive dysfunctions represent a reliable predictor of cognitive decline and may provide insight into the future clinical course of the disease. The implications of these impairments are substantial, impacting patients' ability to plan and make decisions, sustain attention on complex tasks, and manage daily activities.

Looking ahead, executive function measures hold promise as clinical biomarkers in HD. Given that certain executive deficits emerge well before motor onset, sensitive cognitive tests or neurophysiological indicators could aid in earlier diagnosis and help track disease progression in clinical trials. Early implementation of cognitive assessment strategies will therefore be important; routine monitoring of executive performance in gene‐expansion carriers or early‐stage patients may allow clinicians to detect subtle changes and initiate supportive interventions sooner. Another key avenue is the development of targeted approaches to preserve or improve cognitive function. Nonpharmacological interventions such as structured cognitive training programs or rehabilitation focused on executive skills are being explored, aiming to harness neuroplasticity and potentially slow cognitive decline. Meanwhile, although no medications are yet approved specifically for cognitive impairment in HD, research continues to investigate pharmacological options—for example, compounds that enhance neurotransmitter function or protect against neurodegeneration—that might ameliorate executive deficits. By combining early cognitive detection with emerging therapeutic strategies, future clinical management of HD may achieve better preservation of cognitive abilities and, ultimately, improve patient quality of life.

## Author Contributions

**Simone Migliore**: Conceptualization, Methodology, Writing – original draft, Writing – review & editing, Supervision. **Martina Marcaccio**: Data curation, Methodology; **Ilaria Di Pompeo**: Methodology, Data curation. **Massimo Marano**: Writing – review & editing, Conceptualization. **Giuseppe Curcio**: Conceptualization, Writing – original draft, Writing – review & editing, Methodology, Supervision.

## Funding

The authors have nothing to report.

## Ethics Statement

The authors have nothing to report.

## Conflicts of Interest

The authors declare no conflicts of interest.

## Author Agreement

All the authors have seen and approved the final version of the manuscript being submitted. The article is the authors' original work, has not received prior publication and is not under consideration for publication elsewhere.

## Supporting information




**Supporting Information**: brb371238‐sup‐0001‐SuppMat.docx

## Data Availability

All data generated or analyzed during this study are included in this article.
