# Supplementary material for: Executive Impairment in Huntington's Disease: Insights From a Systematic Review of the Literature
Source: Brain Behav. 2026 Jan 31;16(2):e71238. doi: 10.1002/brb3.71238 (PMC12860536; doi:10.1002/brb3.71238)
Supplement: Supplementary file 1 — Supporting Information: brb371238‐sup‐0001‐SuppMat.docx [file BRB3-16-e71238-s001.docx]

**REFERENCES FIGURE 2**

1. D’Aurizio, G.; Migliore, S.; Curcio, G.; Squitieri, F. Safer Attitude to Risky Decision-Making in Premanifest Huntington’s Disease Subjects. Front Psychol 2019, 10, 846, doi:10.3389/fpsyg.2019.00846.

2. Verny, C.; Allain, P.; Prudean, A.; Malinge, M. ‐C.; Gohier, B.; Scherer, C.; Bonneau, D.; Dubas, F.; Le Gall, D. Cognitive Changes in Asymptomatic Carriers of the Huntington Disease Mutation Gene. Eur J Neurol 2007, 14, 1344–1350, doi:10.1111/j.1468-1331.2007.01975.x.

3. Hart, E.P.; Dumas, E.M.; Schoonderbeek, A.; Wolthuis, S.C.; Van Zwet, E.W.; Roos, R.A.C. Motor Dysfunction Influence on Executive Functioning in Manifest and Premanifest Huntington’s Disease. Movement Disorders 2014, 29, 320–326, doi:10.1002/mds.25806.

4. Snowden, J.S.; Craufurd, D.; Thompson, J.; Neary, D. Psychomotor, Executive, and Memory Function in Preclinical Huntington’s Disease. J Clin Exp Neuropsychol 2002, 24, 133–145, doi:10.1076/jcen.24.2.133.998.

5. Carvalho, J.O.; Long, J.D.; Westervelt, H.J.; Smith, M.M.; Bruce, J.M.; Kim, J.-I.; Mills, J.A.; Paulsen, J.S.; The Predict-Hd Investigators And Co The Impact of Oculomotor Functioning on Neuropsychological Performance in Huntington Disease. J Clin Exp Neuropsychol 2016, 38, 217–226, doi:10.1080/13803395.2015.1101054.

6. Paz-Rodríguez, F.; Chávez-Oliveros, M.; Bernal-Pérez, A.; Ochoa-Morales, A.; Martínez-Ruano, L.; Camacho-Molina, A.; Rodríguez-Agudelo, Y. Neuropsychological Performance and Disease Burden in Individuals at Risk of Developing Huntington Disease. Neurologia 2021, 39, 127–134, doi:10.1016/J.NRL.2021.04.015.

7. Larsson, M.U.; Almkvist, O.; Luszcz, M.A.; Wahlin, T.-B.R. Phonemic Fluency Deficits in Asymptomatic Gene Carriers for Huntington’s Disease. Neuropsychology 2008, 22, 596–605, doi:10.1037/0894-4105.22.5.596.

8. Dumas, E.M.; Say, M.J.; Jones, R.; Labuschagne, I.; O’Regan, A.M.; Hart, E.P.; Van Den Bogaard, S.J.A.; Queller, S.; Justo, D.; Coleman, A.; et al. Visual Working Memory Impairment in Premanifest Gene-Carriers and Early Huntington’s Disease. J Huntingtons Dis 2012, 1, 97–106, doi:10.3233/JHD-2012-120010.

9. Reyes, A.; Bartlett, D.M.; Rankin, T.J.; Zaenker, P.; Turner, K.; Teo, W.-P.; Fu, S.C.; Domingos, J.; Georgiou-Karistianis, N.; Ziman, M.; et al. Clinical Determinants of Dual Tasking in People With Premanifest Huntington Disease. Phys Ther 2021, 101, pzab016, doi:10.1093/ptj/pzab016.

10. Horta-Barba, A.; Martinez-Horta, S.; Perez-Perez, J.; Sampedro, F.; De Lucia, N.; De Michele, G.; Salvatore, E.; Kehrer, S.; Priller, J.; Migliore, S.; et al. Arithmetic Word-Problem Solving as Cognitive Marker of Progression in Pre-Manifest and Manifest Huntington’s Disease. J Huntingtons Dis 2021, 10, 459–468, doi:10.3233/JHD-210480.

11. El Haj, M.; Caillaud, M.; Moustafa, A.; Prundean, A.; Scherer, C.; Verny, C.; Allain, P. “Ten Euros Now” Temporal Discounting in Huntington Disease. Neurological Sciences 2023, 44, 2763–2771, doi:10.1007/s10072-023-06775-z.

12. Farrow, M.; Chua, P.; Churchyard, A.; Bradshaw, J.L.; Chiu, E.; Georgiou- Karistianis, N. Proximity to Clinical Onset Influences Motor and Cognitive Performance in Presymptomatic Huntington Disease Gene Carriers. Cognitive and Behavioral Neurology 2006, 19, 208–216, doi:10.1097/01.wnn.0000213914.64772.b6.

13. Farrow, M.; Churchyard, A.; Chua, P.; Bradshaw, J.L.; Chiu, E.; Georgiou- Karistianis, N. Attention, Inhibition, and Proximity to Clinical Onset in Preclinical Mutation Carriers for Huntington’s Disease. J Clin Exp Neuropsychol 2007, 29, 235–246, doi:10.1080/13803390600657693.

14. O’Rourke, J.J.F.; Beglinger, L.J.; Smith, M.M.; Mills, J.; Moser, D.J.; Rowe, K.C.; Langbehn, D.R.; Duff, K.; Stout, J.C.; Harrington, D.L.; et al. The Trail Making Test in Prodromal Huntington Disease: Contributions of Disease Progression to Test Performance. J Clin Exp Neuropsychol 2011, 33, 567–579, doi:10.1080/13803395.2010.541228.

15. Migliore, S.; D’Aurizio, G.; Curcio, G.; Squitieri, F. Task-Switching Abilities in Pre- Manifest Huntington’s Disease Subjects. Parkinsonism Relat Disord 2019, 60, 111–117, doi:10.1016/j.parkreldis.2018.09.007.

16. Jacobs, M.; Hart, E.P.; Miranda, Y.M.; Groeneveld, G.J.; Van Gerven, J.M.A.; Roos, R.A.C. Altered Driving Performance of Symptomatic Huntington’s Disease Gene Carriers in Simulated Road Conditions. Traffic Inj Prev 2018, 19, 708–714, doi:10.1080/15389588.2018.1497796

17. Hennig, B.L.; Kaplan, R.F.; Nowicki, A.E.; Barclay, J.E.; Gertsberg, A.G. We Can Predict When Driving Is No Longer Safe for People Who Have HD Using Standard Neuropsychological Measures. J Huntingtons Dis 2014, 3, 351–353, doi:10.3233/JHD-140125.

18. Devos, H.; Nieuwboer, A.; Vandenberghe, W.; Tant, M.; De Weerdt, W.; Uc, E.Y. On-Road Driving Impairments in Huntington Disease. Neurology 2014, 82, 956– 962, doi:10.1212/WNL.0000000000000220.

19. Devos, H.; Nieuwboer, A.; Tant, M.; De Weerdt, W.; Vandenberghe, W. Determinants of Fitness to Drive in Huntington Disease. Neurology 2012, 79, 1975–1982, doi:10.1212/WNL.0b013e3182735d11.

20. Beglinger, L.J.; Prest, L.; Mills, J.A.; Paulsen, J.S.; Smith, M.M.; Gonzalez‐Alegre, P.; Rowe, K.C.; Nopoulos, P.; Uc, E.Y. Clinical Predictors of Driving Status in Huntington’s Disease. Movement Disorders 2012, 27, 1146–1152, doi:10.1002/mds.25101.

21. Brandt, J.; Inscore, A.B.; Ward, J.; Shpritz, B.; Rosenblatt, A.; Margolis, R.L.; Ross, C.A. Neuropsychological Deficits in Huntington’s Disease Gene Carriers and Correlates of Early “Conversion.” J Neuropsychiatry Clin Neurosci 2008, 20, 466– 472, doi:10.1176/jnp.2008.20.4.466.

22. Watkins, L.H.A.; Rogers, R.D.; Lawrence, A.D.; Sahakian, B.J.; Rosser, A.E.; Robbins, T.W. Impaired Planning but Intact Decision Making in Early Huntington’s Disease: Implications for Specific Fronto-Striatal Pathology. Neuropsychologia 2000, 38, 1112–1125, doi:10.1016/S0028-3932(00)00028-2.

23. Allain, P.; Verny, C.; Aubin, G.; Pinon, K.; Bonneau, D.; Dubas, F.; Gall, D. Le Arithmetic Word-Problem-Solving in Huntington’s Disease. Brain Cogn 2005, 57, 1–3, doi:10.1016/j.bandc.2004.08.010.

24. Solca, F.; Poletti, B.; Zago, S.; Crespi, C.; Sassone, F.; Lafronza, A.; Maraschi, A.M.; Sassone, J.; Silani, V.; Ciammola, A. Counterfactual Thinking Deficit in Huntington’s Disease. PLoS One 2015, 10, e0126773, doi:10.1371/journal.pone.0126773.

25. Ho, A.K.; Sahakian, B.J.; Brown, R.G.; Barker, R.A.; Hodges, J.R.; Ane, M.-N.; Snowden, J.; Thompson, J.; Esmonde, T.; Gentry, R.; et al. Profile of Cognitive Progression in Early Huntington’s Disease. Neurology 2003, 61, 1702–1706, doi:10.1212/01.WNL.0000098878.47789.BD.

26. Lemiere, J.; Decruyenaere, M.; Evers-Kiebooms, G.; Vandenbussche, E.; Dom, R. Cognitive Changes in Patients with Huntington?S Disease (HD) and Asymptomatic Carriers of the HD Mutation: A Longitudinal Follow?Up Study. J Neurol 2004, 251, doi:10.1007/s00415-004-0461-9.

27. Bachoud–Lévi, A. –C.; Maison, P.; Bartolomeo, P.; Boissé, M. –F.; Dalla Barba, G.; Ergis, A. –M.; Baudic, S.; Degos, J. –D.; Cesaro, P.; Peschanski, M. Retest Effects and Cognitive Decline in Longitudinal Follow-up of Patients with Early HD. Neurology 2001, 56, 1052–1058, doi:10.1212/WNL.56.8.1052.

28. Couette, M.; Bachoud-Levi, A.-C.; Brugieres, P.; Sieroff, E.; Bartolomeo, P. Orienting of Spatial Attention in Huntington’s Disease. Neuropsychologia 2008, 46, 1391–1400, doi:10.1016/j.neuropsychologia.2007.12.017.

29. Georgiou-Karistianis, N.; Farrow, M.; Churchyard, A. Deficits in Selective Attention in Symptomatic Huntington Disease: Assessment Using an Attentional Blink Paradigm. Cogn Behav Neurol 2012, 25.

**REFERENCES FIGURE 3**

1. Beste, C.; Ness, V.; Lukas, C.; Hoffmann, R.; Stüwe, S.; Falkenstein, M.; Saft, C. Mechanisms Mediating Parallel Action Monitoring in Fronto-Striatal Circuits. Neuroimage 2012, 62, 137–146, doi:10.1016/j.neuroimage.2012.05.019.

2. Hart, E.P.; Dumas, E.M.; Reijntjes, R.H.A.M.; Hiele, K.; Bogaard, S.J.A.; Middelkoop, H.A.M.; Roos, R.A.C.; Dijk, J.G. Deficient Sustained Attention to Response Task and P300 Characteristics in Early Huntington’s Disease. J Neurol 2012, 259, 1191–1198, doi:10.1007/s00415-011-6334-0.

3. Beste, C.; Ness, V.; Falkenstein, M.; Saft, C. On the Role of Fronto-Striatal Neural Synchronization Processes for Response Inhibition—Evidence from ERP Phase- Synchronization Analyses in Pre-Manifest Huntington’s Disease Gene Mutation Carriers. Neuropsychologia 2011, 49, 3484–3493, doi:10.1016/j.neuropsychologia.2011.08.024.

4. Beste, C.; Stock, A.K.; Ness, V.; Hoffmann, R.; Saft, C. Evidence for Divergent Effects of Neurodegeneration in Huntington’s Disease on Attentional Selection and Neural Plasticity: Implications for Excitotoxicity. Brain Struct Funct 2015, 220, 1437–1447, doi:10.1007/S00429-014-0735-7.

5. Jurgens, C.K.; van der Hiele, K.; Reijntjes, R.H.A.M.; van de Wiel, L.; Witjes-Ané, M.N.W.; van der Grond, J.; Roos, R.A.C.; Middelkoop, H.A.M.; van Dijk, J.G. Basal Ganglia Volume Is Strongly Related to P3 Event-Related Potential in Premanifest Huntington’s Disease. Eur J Neurol 2011, 18, 1105–1108, doi:10.1111/J.1468- 1331.2010.03309.X.

6. Beste, C.; Saft, C.; Andrich, J.; Gold, R.; Falkenstein, M. Stimulus-Response Compatibility in Huntington’s Disease: A Cognitive-Neurophysiological Analysis. J Neurophysiol 2008, 99, 1213–1223, doi:10.1152/JN.01152.2007.

7. Beste, C.; Saft, C.; Güntürkün, O.; Falkenstein, M. Increased Cognitive Functioning in Symptomatic Huntington’s Disease As Revealed by Behavioral and Event-Related Potential Indices of Auditory Sensory Memory and Attention. The Journal of Neuroscience 2008, 28, 11695–11702, doi:10.1523/JNEUROSCI.2659-08.2008.

8. Cheng, C.-H.; Soong, B.-W.; Hsu, W.-Y.; Lin, Y.-Y. Reduced Automatic Frontal Response to Auditory Deviance in Huntington’s Disease as Indexed by Magnetic Mismatch Negativity. Journal of Clinical Neuroscience 2014, 21, 1773–1778, doi:10.1016/j.jocn.2014.01.019.

9. Wolf, R.C.; Vasic, N.; Schönfeldt‐Lecuona, C.; Ecker, D.; Landwehrmeyer, G.B. Cortical Dysfunction in Patients with Huntington’s Disease during Working Memory Performance. Hum Brain Mapp 2009, 30, 327–339, doi:10.1002/hbm.20502.

10. Aron, A.R.; Watkins, L.; Sahakian, B.J.; Monsell, S.; Barker, R.A.; Robbins, T.W. Task-Set Switching Deficits in Early-Stage Huntington’s Disease: Implications for Basal Ganglia Function. J Cogn Neurosci 2003, 15, 629–642, doi:10.1162/089892903322307357.

11. Kassubek, J.; Juengling, F.D.; Ecker, D.; Landwehrmeyer, G.B. Thalamic Atrophy in Huntington’s Disease Co-Varies with Cognitive Performance: A Morphometric MRI Analysis. Cereb Cortex 2005, 15, 846–853, doi:10.1093/CERCOR/BHH185.

12. Peinemann, A.; Schuller, S.; Pohl, C.; Jahn, T.; Weindl, A.; Kassubek, J. Executive Dysfunction in Early Stages of Huntington’s Disease Is Associated with Striatal and Insular Atrophy: A Neuropsychological and Voxel-Based Morphometric Study. J Neurol Sci 2005, 239, 11–19, doi:10.1016/J.JNS.2005.07.007.

13. Vaca-Palomares, I.; Coe, B.C.; Brien, D.C.; Campos-Romo, A.; Munoz, D.P.; Fernandez-Ruiz, J. Voluntary Saccade Inhibition Deficits Correlate with Extended White-Matter Cortico-Basal Atrophy in Huntington’s Disease. Neuroimage Clin 2017, 15, 502–512, doi:10.1016/j.nicl.2017.06.007.

14. Martinez-Horta, S.; Sampedro, F.; Horta-Barba, A.; Perez-Perez, J.; Pagonabarraga, J.; Gomez-Anson, B.; Kulisevsky, J. Structural Brain Correlates of Dementia in Huntington’s Disease. Neuroimage Clin 2020, 28, 102415, doi:10.1016/j.nicl.2020.102415.

15. Casella, C.; Kleban, E.; Rosser, A.E.; Coulthard, E.; Rickards, H.; Fasano, F.; Metzler-Baddeley, C.; Jones, D.K. Multi-Compartment Analysis of the Complex Gradient-Echo Signal Quantifies Myelin Breakdown in Premanifest Huntington’s Disease. Neuroimage Clin 2021, 30, 102658, doi:10.1016/j.nicl.2021.102658.

16. Kim, H.; Kim, J.; Possin, K.L.; Winer, J.; Geschwind, M.D.; Xu, D.; Hess, C.P. Surface-Based Morphometry Reveals Caudate Subnuclear Structural Damage in Patients with Premotor Huntington Disease. Brain Imaging Behav 2017, 11, 1365–1372, doi:10.1007/s11682-016-9616-4.

17. Soloveva, M. V; Jamadar, S.D.; Velakoulis, D.; Poudel, G.; Georgiou-Karistianis, N. Brain Compensation during Visuospatial Working Memory in Premanifest Huntington’s Disease. Neuropsychologia 2020, 136, 107262, doi:10.1016/j.neuropsychologia.2019.107262.

18. Possin, K.L.; Kim, H.; Geschwind, M.D.; Moskowitz, T.; Johnson, E.T.; Sha, S.J.; Apple, A.; Xu, D.; Miller, B.L.; Finkbeiner, S.; et al. Egocentric and Allocentric Visuospatial Working Memory in Premotor Huntington’s Disease: A Double Dissociation with Caudate and Hippocampal Volumes. Neuropsychologia 2017, 101, 57–64, doi:10.1016/J.NEUROPSYCHOLOGIA.2017.04.022.

19. Papp, K. V; Snyder, P.J.; Mills, J.A.; Duff, K.; Westervelt, H.J.; Long, J.D.; Lourens, S.; Paulsen, J.S. Measuring Executive Dysfunction Longitudinally and in Relation to Genetic Burden, Brain Volumetrics, and Depression in Prodromal Huntington Disease. Archives of Clinical Neuropsychology 2013, 28, 156–168, doi:10.1093/arclin/acs105.

20. Matsui, J.T.; Vaidya, J.G.; Johnson, H.J.; Magnotta, V.A.; Long, J.D.; Mills, J.A.; Lowe, M.J.; Sakaie, K.E.; Rao, S.M.; Smith, M.M.; et al. Diffusion Weighted Imaging of Prefrontal Cortex in Prodromal Huntington’s Disease. Hum Brain Mapp 2014, 35, 1562–1573, doi:10.1002/hbm.22273.

21. Matsui, J.T.; Vaidya, J.G.; Wassermann, D.; Kim, R.E.; Magnotta, V.A.; Johnson, H.J.; Paulsen, J.S.; Isabella De Soriano; Shadrick, C.; Miller, A.; et al. Prefrontal Cortex White Matter Tracts in Prodromal Huntington Disease. Hum Brain Mapp 2015, 36, 3717–3732, doi:10.1002/HBM.22835.

22. Wolf, R.C.; Sambataro, F.; Vasic, N.; Schönfeldt-Lecuona, C.; Ecker, D.; Landwehrmeyer, B. Altered Frontostriatal Coupling in Pre-Manifest Huntington’s Disease: Effects of Increasing Cognitive Load. Eur J Neurol 2008, 15, 1180–1190, doi:10.1111/J.1468-1331.2008.02253.X.

23. Wolf, R.C.; Sambataro, F.; Vasic, N.; Schönfeldt-Lecuona, C.; Ecker, D.; Landwehrmeyer, B. Aberrant Connectivity of Lateral Prefrontal Networks in Presymptomatic Huntington’s Disease. Exp Neurol 2008, 213, 137–144, doi:10.1016/j.expneurol.2008.05.017.

24. Wolf, R.C.; Sambataro, F.; Vasic, N.; Wolf, N.D.; Thomann, P.A.; Landwehrmeyer, G.B.; Orth, M. Longitudinal Functional Magnetic Resonance Imaging of Cognition in Preclinical Huntington’s Disease. Exp Neurol 2011, 231, 214–222, doi:10.1016/j.expneurol.2011.06.011.

25. Wolf, R.C.; Grön, G.; Sambataro, F.; Vasic, N.; Wolf, N.D.; Thomann, P.A.; Saft, C.; Landwehrmeyer, G.B.; Orth, M. Brain Activation and Functional Connectivity in Premanifest Huntington’s Disease during States of Intrinsic and Phasic Alertness. Hum Brain Mapp 2012, 33, 2161–2173, doi:10.1002/hbm.21348.

26. Harrington, D.L.; Liu, D.; Smith, M.M.; Mills, J.A.; Long, J.D.; Aylward, E.H.; Paulsen, J.S.; PREDICT‐HD Investigators Coordinators of the Huntington Study Group Neuroanatomical Correlates of Cognitive Functioning in Prodromal Huntington Disease. Brain Behav 2014, 4, 29–40, doi:10.1002/brb3.185.

27. Harrington, D.L.; Rubinov, M.; Durgerian, S.; Mourany, L.; Reece, C.; Koenig, K.; Bullmore, E.; Long, J.D.; Paulsen, J.S.; Rao, S.M. Network Topology and Functional Connectivity Disturbances Precede the Onset of Huntington’s Disease. Brain 2015, 138, 2332–2346, doi:10.1093/BRAIN/AWV145.

28. Poudel, G.R.; Stout, J.C.; Domínguez D, J.F.; Gray, M.A.; Salmon, L.; Churchyard, A.; Chua, P.; Borowsky, B.; Egan, G.F.; Georgiou-Karistianis, N. Functional Changes during Working Memory in Huntington’s Disease: 30-Month Longitudinal Data from the IMAGE-HD Study. Brain Struct Funct 2015, 220, 501– 512, doi:10.1007/S00429-013-0670-Z.

29. Langley, C.; Gregory, S.; Osborne-Crowley, K.; O’Callaghan, C.; Zeun, P.; Lowe, J.; Johnson, E.B.; Papoutsi, M.; Scahill, R.I.; Rees, G.; et al. Fronto-Striatal Circuits for Cognitive Flexibility in Far from Onset Huntington’s Disease: Evidence from the Young Adult Study. J Neurol Neurosurg Psychiatry 2021, 92, 143–149, doi:10.1136/JNNP-2020-324104.

30. Wolf, R.C.; Sambataro, F.; Vasic, N.; Wolf, N.D.; Thomann, P.A.; Saft, C.; Landwehrmeyer, G.B.; Orth, M. Default-Mode Network Changes in Preclinical Huntington’s Disease. Exp Neurol 2012, 237, 191–198, doi:10.1016/j.expneurol.2012.06.014.

31. Wolf, R.C.; Sambataro, F.; Vasic, N.; Wolf, N.D.; Thomann, P.A.; Landwehrmeyer, G.B.; Orth, M. Longitudinal Task-Negative Network Analyses in Preclinical Huntington’s Disease. Eur Arch Psychiatry Clin Neurosci 2014, 264, 493–505, doi:10.1007/s00406-013-0447-7.

32. Unschuld, P.G.; Liu, X.; Shanahan, M.; Margolis, R.L.; Bassett, S.S.; Brandt, J.; Schretlen, D.J.; Redgrave, G.W.; Hua, J.; Hock, C.; et al. Prefrontal Executive Function Associated Coupling Relates to Huntington’s Disease Stage. Cortex 2013, 49, 2661–2673, doi:10.1016/j.cortex.2013.05.015.

33. Pavese, N.; Politis, M.; Tai, Y.F.; Barker, R.A.; Tabrizi, S.J.; Mason, S.L.; Brooks, D.J.; Piccini, P. Cortical Dopamine Dysfunction in Symptomatic and Premanifest Huntington’s Disease Gene Carriers. Neurobiol Dis 2010, 37, 356–361, doi:10.1016/j.nbd.2009.10.015.
